# Porous flower-like superstructures based on self-assembled colloidal quantum dots for sensing

**DOI:** 10.1038/s41598-018-36250-1

**Published:** 2019-01-24

**Authors:** Evgeniia A. Stepanidenko, Yulia A. Gromova, Tatiana K. Kormilina, Sergei A. Cherevkov, Danil A. Kurshanov, Aliaksei Dubavik, Mikhail A. Baranov, Oleg S. Medvedev, Anatoly V. Fedorov, Yurii K. Gun’ko, Elena V. Ushakova, Alexander V. Baranov

**Affiliations:** 1 ITMO University, Saint Petersburg, 197101 Russia; 2 Saint-Petersburg State University, Saint Petersburg, 199034 Russia; 3 School of Chemistry and CRANN, Trinity College Dublin, Dublin, 2 Ireland

## Abstract

Quantum dots (QDs) have been envisaged as very promising materials for the development of advanced optical sensors. Here we report a new highly porous luminescent material based on colloidal QDs for potential applications in optical sensing devices. Bulk flower-like porous structures with sizes of hundreds of microns have been produced by slow destabilization of QD solution in the presence of a non-solvent vapor. The porous highly luminescent material was formed from CdSe QDs using the approach of non-solvent destabilization. This material demonstrated a 4-fold decrease in PL signal in the presence of the ammonia vapor. The relationship between the destabilization rate of QDs in solution and the resulting morphology of structural elements has been established. The proposed model of bulk porous flower-like nanostructured material fabrication can be applied to nanoparticles of different nature combining their unique properties. This research opens up a new approach to design novel multi-component composite materials enabling potential performance improvements of various photonic devices.

## Introduction

The development of colloidal synthesis has opened new opportunities to produce colloidal semiconducting nanocrystals with the desired and controlled optical parameters which can be easily incorporated into various dielectric and conductive matrixes, including flexible, substrates^1,2^. In contrast to semiconductor materials of macro- and micro- sizes, colloidal nanocrystals (NCs) have a number of advantages such as: the possibility of fine tuning of various physical parameters, high values of quantum yields (QYs), insignificant heat losses due to low electron-phonon interaction, and relatively low production costs^3^. By combining unique properties of nanoparticles (NPs), it is possible to create new multicomponent nanostructured materials with well controlled properties^4^.

Colloidal quantum dots (QDs) with sizes up to 10 nm are promising candidates for the development of novel functional materials^1,5^. The advantages of such nanocrystals include: a tunable energy spectrum, a narrow luminescence band, and high QY. For instance, QDs of mixed chemical composition (alloyed QDs) attract much attention due to their higher photochemical and chemical stability comparing to standard binary semiconductor nanocrystals along with relatively large PL lifetimes up to 100 ns^6,7^. The use of such nanostructured materials enables to produce high-efficiency light emitting sources with unique performance parameters that cannot be achieved for “classical” semiconductors. Such luminescent nanoparticles are relevant for various applications^8^, such as optical encoding and marking^9^, technologies for improving the quality of displays, developing luminescent markers for biological and medical research^10–13^, including a detecting malignant tumors, and agents for photodynamic therapy in strongly scattering media^14^. Emitting sources with possibility of random lasing can also be used as active elements in advanced photonic devices^15^ and integrated optical circuits^8^. Another field of QD applications is the development of sensing materials based on QD^16^ due to their high value of surface area to volume ratio, which is the substantial parameter for the improving the sensitivity.

In general, for sensory devices fabrication the materials with larger adsorption surface area, which can be achieved in those with porous morphology, are utilized. There are many examples of porous materials including: porous glasses^17^, aerogels^18^, materials formed by a template assembly^19^, metal-organic frameworks^20,21^ and “flower-like” structures^22^. To date, the most of “flower-like” structures are mainly derived from metal oxides, for instance copper^23–26^ or zinc oxides^22,27–29^.

To date, the scientific community has several challenges to improve sensing materials. Among them there are an improvement of response and sensitivity, reducing of operating temperature, increasing of stability and etc. One of the urgent tasks for researchers is the development of multicomponent porous material combining special/extra properties of each component. The using of nanostructures as consisting components is one of the ways to achieve the above-mentioned improved performance parameters of sensing materials^23–30^. Addition of graphene oxide^31,32^, doping by metal NPs, Au^33^ and Ag^34^, the bulk mesoporous materials are used to achieve good stability and reproducibility, high selectivity and to decrease operating temperature of sensory devices. Many works are devoted to the development of nanostructured sensing materials containing mixtures of nanoparticles with different shapes^24,30,35^. Along with this, copper and zinc doped SnO_2_ nanocrystals are found their application to improvement of the response of mesoporous sensing material^36^.

At the same time, the existing sensors based on metal oxides possess some disadvantages, for instance, a high operating temperature. In addition, most of these sensors are based on an electrical response, which complicates the technological process: the need of deposition on conductive substrates, circuit design, making contacts, etc. Thus, sensors based on optical responses are the more attractive, since they are compact, lightweight and convenient. Furthermore, such photoluminescent sensors have the fastest response time and the best sensitivity. Further development of optical sensors is anticipated by combining the unique optical properties of QDs and the formation of highly porous materials based on them.

In this work, for the first time to our knowledge, we report an approach to produce structured porous materials based on colloidal Cd-based quantum dots. The new photonic porous materials have been tested as luminescent sensors of ammonia vapors.

## Results and Discussion

Porous materials based on nanoparticles can be prepared by the template method^37–39^ and bottom up self-assembly method^40–43^. The latter method is more flexible and allows fabricating the nanostructured materials from nanoparticles of various types on different substrates. In this work the destabilization of a nanoparticle colloidal solution was applied to QD superstructure formation. Alloyed Cd_1−*x*_Zn_*x*_Se_1−*y*_S_*y*_/ZnS QDs with an average size of 7.0 ± 1.0 nm were used as model nanoparticles for the initial development of the protocol for the preparation of nanostructured material with increased porosity. The indexes in the formula of alloyed QDs show the molar ratio between elements in alloyed QDs. Core type CdSe QDs with mean size of 4.0 ± 0.5 nm were then used for further experiment on sensing properties of the nanostructured material and their dependence on its morphology. To derive the best synthetic protocol for formation of suitable material 4 different methods of destabilization of colloidal solution were used: slow evaporation^44^ (sample QD1), slow evaporation in the non-solvent vapor presence^45^ (sample QD2), destabilization by non-solvent^46^ (sample QD3), destabilization by addition of the ligand molecules^47^ (sample QD4). Also, a reference sample was prepared by setting a Si substrate in a colloidal solution for 1 month, designated further as QD5. Thus, 5 samples were produced from Cd_1−*x*_Zn_*x*_Se_1−*y*_S_*y*_/ZnS QDs assembled on inclined Si substrates. For testing the sensor performance of samples with different morphology, superstructures based on CdSe QD were prepared by destabilization in the presence of non-solvent (vapor and in solution). All details are given in Methods and Supporting Information (S1).

### Morphology of structures based on alloyed Cd_1−*x*_Zn_*x*_Se_1−*y*_S_*y*_/ZnS QDs

**Figure 1:**
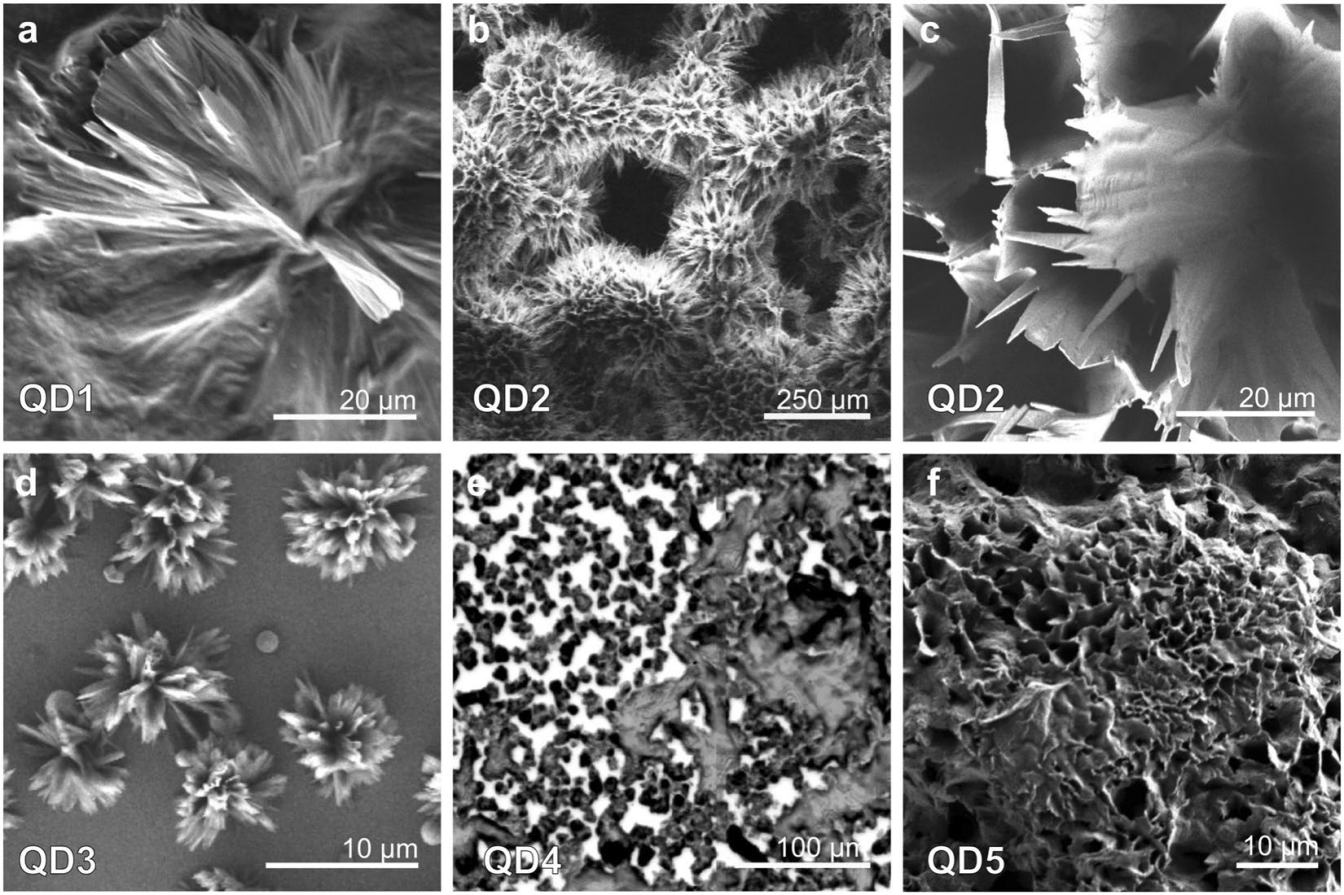
Morphology of superstructure from alloyed QDs: (**a**) QD1; (**b**,**c**) QD2; (**d**) QD3; (**e**) QD4; (**f**) QD5.

**Figure 2:**
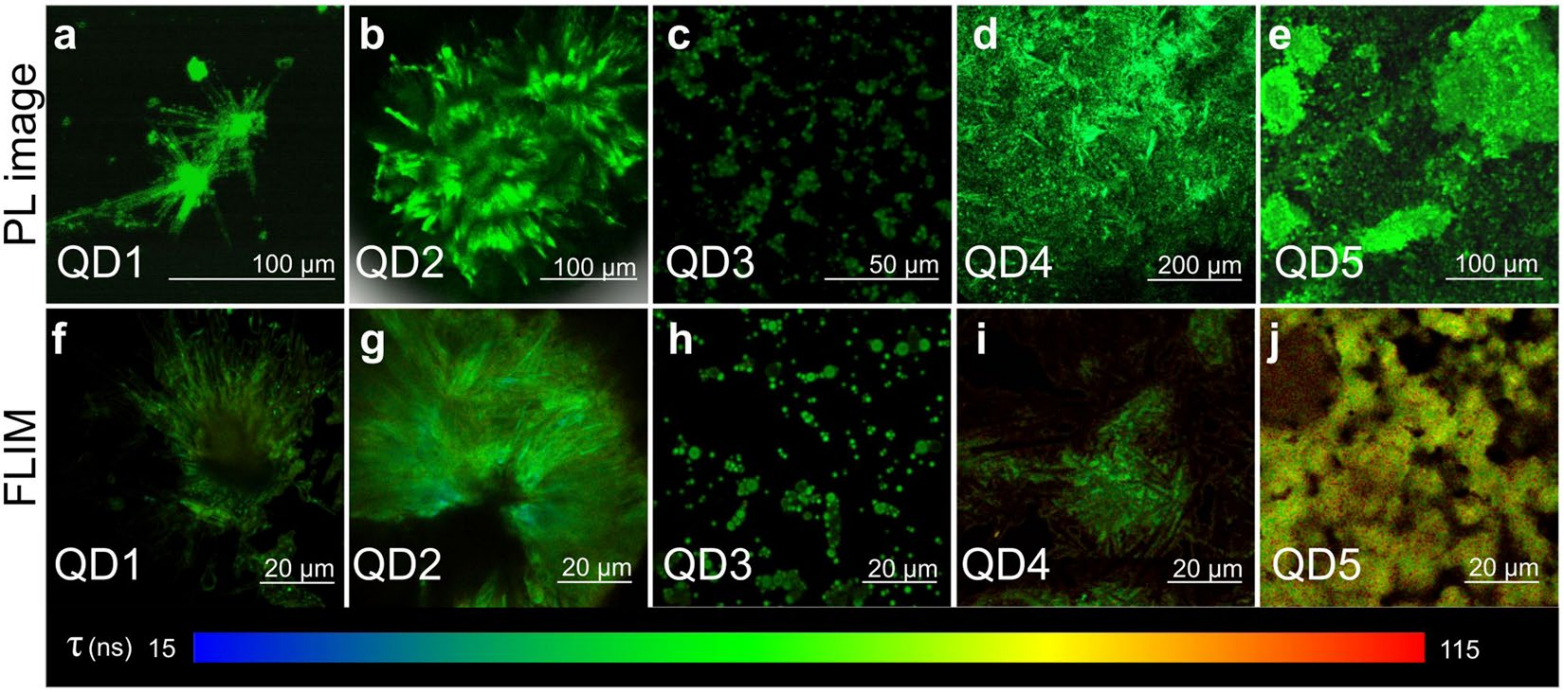
Typical optical PL images (**a**–**e**) and FLIM (**f**–**j**) of superstructures based on alloyed QDs: (**a**,**f**) QD1, (**b**,**g**) QD2, (**c**,**h**) QD3, (**d**,**i**) QD4, (**e**,**j**) QD5 and time scale for FLIM.

Scanning electron microscopy (SEM) images of typical superstructures formed by alloyed QDs are presented in Fig. 1. QD1 superstructures include the bulk porous structure consisting of large flat spiky flowers with the size of 40–100 *μ*m and needles with the size of 20–40 *μ*m which cover the Si substrate (Figs 1a and S2 in Supporting Information). Typical morphology of QD2 is a bulk porous structure consisting of large spiky flowers, as shown at Figs 1b,c and S3 in Supporting Information, with various diameters basically from 200 to 500 *μ*m which was formed on the lower part of Si substrate. Analysis of SEM images showed that the mean pore size is 26.5 *μ*m (statistics considered at least 35 pores). In QD3 superstructures with various shapes were found: spikes, flowers, smooth spheres. The sizes of spiky flowers (see Figs 1d and S4 in Supporting Information) were estimated to be 6.7–20.7 *μ*m. The estimated average pore size is 0.3–0.7 *μ*m. Globular microflowers and small flabellate structures are located in the upper-middle part of the Si substrate with sizes varying from 3 to 6 *μ*m. Small smooth spheres with diameters of 0.5 to 2.8 *μ*m occupy a large area of the middle part of the substrate. The QD4 sample was obtained after the temperature decrease. The excess of oleic acid prevented the SEM analysis due to following aspects: the electron beams destroy organics in the sample affecting their morphology, and resulting images have low contrast. Therefore, the morphology of QD4 was explored by means of optical microscopy in reflected light (Figs 1e and S5 in Supporting Information). Bulk structures with size of hundreds microns were found on the substrate. Also the porous needle-like structure covering the substrate, which is similar to those QD1 samples, was observed. At last, the reference sample (QD5) contained porous amorphous structures with an average pore size 1.9 *μ*m covering all substrate similar to those obtained in QD4 sample. At the bottom of the substrate as in the case of QD1 and QD2 samples the bulk porous structure has been grown (Figs 1f and S6 in Supporting Information), however, it did not contain well-defined structural elements. All the structures were formed by the QDs, which was confirmed by the elemental analysis (S7 in Supporting Information) and luminescent analysis of the samples, shown in Fig. 2.

Analysis of the morphology of the QD4 and QD5 samples does not reveal any ordered structures. It can be explained by the lack of chemical destabilization of the QD solution. In cases of QD4 and QD5 QDs deposition on the substrate from solution in random order and the formation of an amorphous structure occurred. These QD solutions were almost stable, since they were prepared in conditions which prevent solvent evaporation. QD4 sample was produced after decreasing the temperature and the deposition was caused by the crystallization of oleic acid in solution. The formation of the reference QD5 sample was very slow. We assume that QDs precipitated on the substrate under gravitational forces.

The analysis of ordered superstructures’ morphology showed that there is dependence in set of samples QD1–QD3 related to the rate of colloidal solution destabilization. A model of the interaction of nanoparticles in colloidal solution during a destabilization was offered in our previous work^43^. Following the proposed model, nanocrystals move chaotically in the colloidal solution, their movement in this environment can be described using the translational (*v*_*trans*_) and orientational (*v*_*orient*_) diffusion rates, and the rate of destabilization of the solution (*v*_*dest*_) for the nanoparticle assembly by destabilization of colloidal solution. In the set QD1–QD3 samples v_*dest*_ increases. According to this model, the spiky flowers will form at high *v*_*orient*_/*v*_*trans*_ ratio due to the oriented attachment mechanism, which was observed in QD1 and QD2. However, these parameters influence the process of superstructure formation in nonlinear way: in the set QD1–QD3 the type of morphology changes from spiky structures to smooth spheres that is in agreement with *v*_*orient*_/*v*_*trans*_ ratio, while size of structural elements is the largest for the QD2 sample. Thus, size of superstructures is also influenced by the presence of non-solvent. Let us consider this aspect in more detail.

In the case of QD1, the quantum dots started to self-assemble on the substrate (or on the walls of the vial), so the materials were formed as flat spiky structures and their growing occurred along the substrate. In QD2 and QD3 samples, structures began to form in solution, followed by settling on the substrate. This is caused by the destabilization of the solution in the presence of non-solvent, which in turn affects the chemical interactions of QD in solution. Thereby, in QD3 separate structural elements (globular flowers and spiky flowers) were observed. In QD2, after the settling, the structures continued to grow because of the slower rate of destabilization of the solution compared with QD3 and presence of available QDs in the vicinity of the superstructure. Thus, the QD1 preparation method resulting in flat irregular structures is not suitable for the application in sensor fabrication. By contrast to that the QD2 preparation method leads to the formation of uniform porous structural elements with mean size of hundreds of microns. Therefore, this nanostructured material has a large surface area, which, as mentioned before, is one of the significant factors in developing highly efficient sensor materials.

### Optical properties of structures based on alloyed Cd_1−*x*_Zn_*x*_Se_1−*y*_S_*y*_/ZnS QDs

We have investigated the optical parameters of the obtained structures. Photoluminescent (PL) images together with fluorescence lifetime images (FLIM) are presented in Fig. 2.

The formed superstructures in all samples are luminescent, confirming that these superstructures consist of alloyed QDs. The morphology of samples observed in PL images and FLIM is similar to that obtained by electron microscopy. Spiky flowers structures were observed in QD1 and QD2. Since the sizes of structural elements in QD3 (globular flowers and smooth spheres) are less than several microns, they cannot be distinguished in optical images. In PL images of QD4 small needles forming an amorphous structure can be observed. In reference sample QD5, amorphous QD ensembles covering the substrate were found. PL spectra of the samples compared to initial QD solution and their PL decay curves are presented in Supporting Information (S8). The optical and morphological parameters are shown in Table 1.

**Table 1:** Optical parameters of the samples formed by alloyed QDs together with their size and morphology.

Sample	Superstructure morphology	Size, *μ*m	Estimated average pore size, *μ*m	PL peak position, nm	FWHM, nm	Average PL lifetime, ns
Alloyed QD solution	—	—	—	535	30	30.9
QD1	Spiky flowers	40–100	—	529	37	21.7
	spikes or needles	20–40				
QD2	Spiky flowers	150–550	26.5	534	49	35.7
	spikes or needles	25–140				
QD3	Spiky flowers	21	0.3–0.7	534	30	17.8
	Globular flowers	3–12				
	Smooth spheres	0.5–2.8				
QD4	Porous structure	>100	—	531	33	29.6
QD5	Porous structure	>100	1.9	532	32	55.7

For all samples the slight changes in PL peak position and full width at the half maximum (FWHM) were observed: the band is blue-shifted and broadened. PL decay of QDs was fitted by a multiexponential function: $I(t)={I}_{0}+{\sum }_{i}\,{{\rm{A}}}_{{\rm{i}}}{\tau }_{i}$ with average PL lifetime calculated as ${\tau }_{av}=\frac{{\sum }_{i}\,{{\rm{A}}}_{{\rm{i}}}{\tau }_{i}^{2}}{{\sum }_{i}\,{{\rm{A}}}_{{\rm{i}}}{\tau }_{i}}$, where A_i_ and τ_i_ is amplitude and lifetime, respectively, of i component, and I_0_ is the background. In the case of alloyed QDs PL decay contains 3 components. A decreasing of the shorter (2 short components) PL lifetimes for all samples was observed, as it is presented in Table S8 in Supporting Information (S8). This results in reducing of the average PL lifetime (*τ*_*av*_) for samples QD1 and QD3. For QD4 the average PL lifetime corresponded to that for the colloidal solution, with amplitude redistribution between components. For QD2 and QD5 an increase in the amplitude for long lifetime component was observed. This behavior can be explained taking into account the QD interaction within the structure formed, namely reabsorbing and reemission.

The comparison of the optical properties with sample morphology suggests the use of QD3 preparation method for fabrication of microemitters with the possibility of random lasing. At the same time, QD2 preparation method is perspective for applications in sensor fabrication, since obtained porous superstructures have a large surface area and QDs in these structures retain good optical parameters.

### Application as sensor of porous structures formed by CdSe QDs

Chemical interactions of QDs with certain compounds can result in changes in their optical properties. In particular, the interaction of semiconductor CdSe QDs with vapors containing nitrogen results in luminescence quenching correlated with the concentration of vapors^16^. Orlova A. *et al*.^48^ demonstrated that the exposure of the porous glass samples with embedded CdSe/ZnS QDs to ammonia vapor results in effective quenching of the QD PL and a reduction in the average PL decay time due to the formation of QD/ammonia complexes on the surface of the CdSe/ZnS QDs. The transition from isolated QDs to superstructures based on them could lead to an enhancement of the sensor performance.

**Figure 3:**
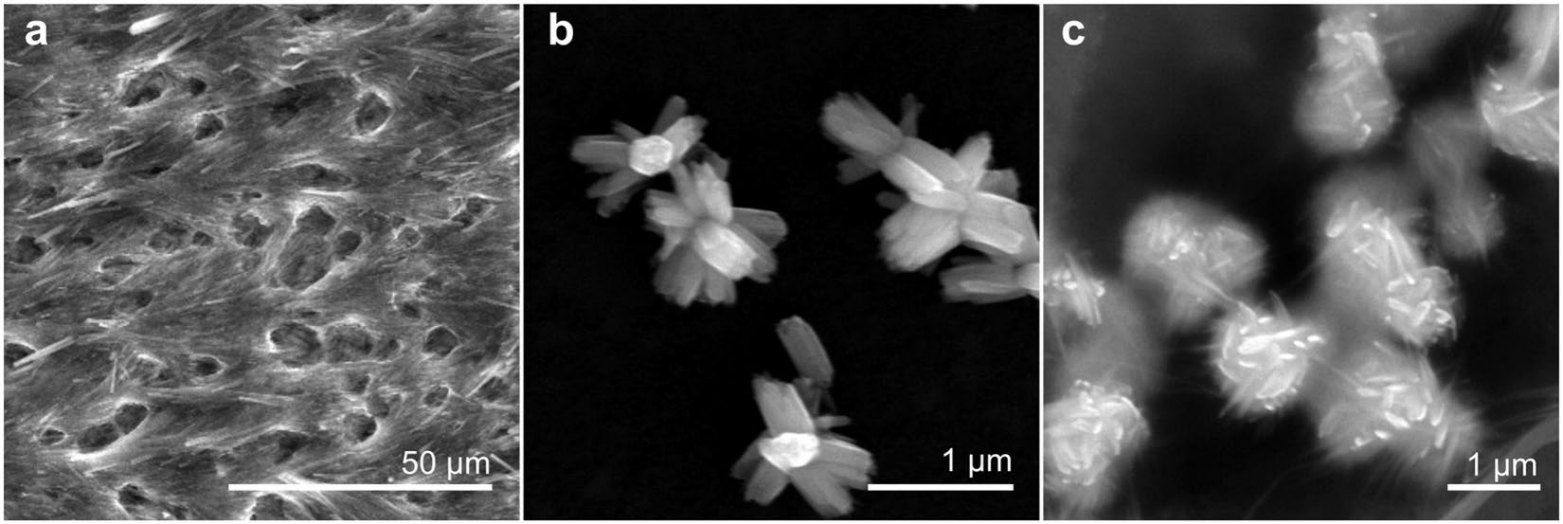
SEM images of superstructures from CdSe QDs: (**a**) porous structure of C1, (**b**) flower-like structures of C2, (**c**) globular flowers of C2.

As mentioned before, the QD2 possesses porous superstructures with large surface area, therefore the QD2 sample was tested for the ammonia vapor detection. However, no evident changes in PL signal were observed, as it is shown in Supporting Information (S9). This behavior can be explained by the alloyed structure of QDs, which leads to the strong localization of exciton in the QD core and eliminates the influence of surface charge traps on QD PL properties. Based on this assumption, we investigated sensor properties of superstructure based on CdSe core type QDs and obtained by the suitable methods for further applications. To examine sensor properties of described porous structures, two samples (samples C1 and C2) from CdSe QD solution with 1.7 × 10^−7^ M concentration were prepared. The QD2 and QD3 methods of superstructure formation have been chosen as more appropriate for further investigation of QD superstructure’ sensing properties. This selection based on the unique flower-like morphology of QD2 and QD3 samples which possesses a large surface area required for sensory applications. The difference in sizes of similar porous structures in QD2 and QD3 and, consequently, in the value of the surface area made it possible to compare sensing properties in dependence on this parameter. The C1 sample was formed by the QD2 formation method using destabilization by the non-solvent vapor. C2 was formed by the QD3 formation method using destabilization by the non-solvent solution with a buffer layer. Si substrates are placed at the vial bottom. Details of procedure are given in Methods section. SEM images of C1 and C2 are presented in Fig. 3. The morphology of the samples C1 and C2 is similar with one of QD2 and QD3, respectively. The sample C1 has a porous structure covering whole substrate and consists of needles and micropores. In sample C2, as in QD3, we found the presence of flower-like structures and globular flowers with the size of 0.5 *μ*m for petals and 0.7–1.4 *μ*m for globular flowers. More SEM images and optical properties of the samples are presented in Supporting Information (S10 and S11).

**Figure 4:**
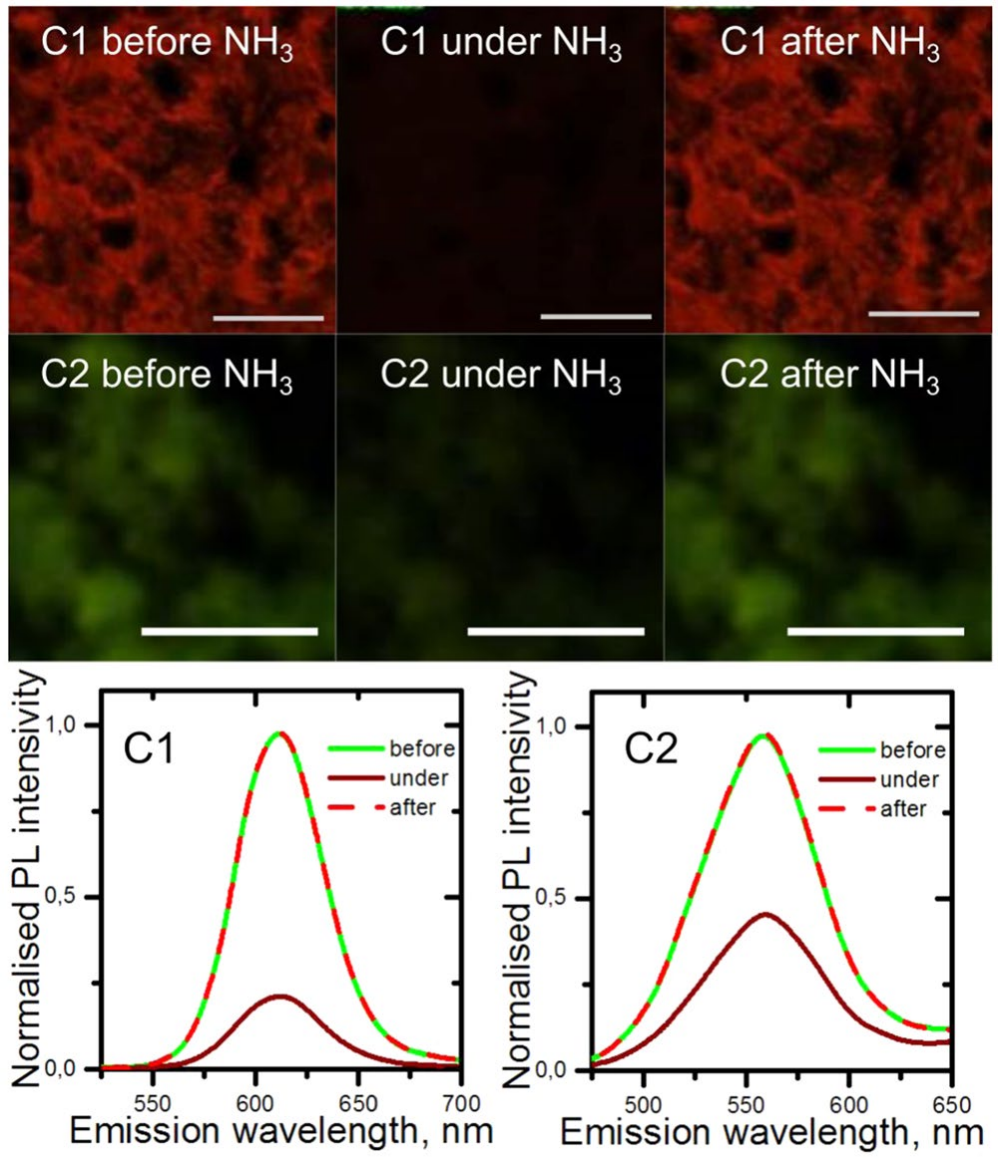
PL images of C1 (upper panel) and C2 (middle panel) samples under NH_3_ treatment and corresponding PL spectra (bottom panel). Scale bar: 50 *μ*m.

We have investigated the change in the optical response of superstructures in the presence of ammonia vapors. PL images together with PL spectra for C1 and C2 samples treated by ammonia vapor are presented in Fig. 4.

The PL intensity sharply decreased in the presence of NH_3_ for both samples. As expected, the C1 demonstrate better sensoring ability than C2, because of the specific morphology – the larger surface area. The PL intensity rapidly decreased by a factor of 4 compared to 2 for C2 when ammonia vapors are injected into the vicinity of the porous superstructure. The reason of QDs PL quenching by ammonia is not straightforward. The low energy of highest occupied molecular orbital (HOMO)^49^ in ammonia makes electron transfer from ammonia to QD prohibited as well as hole transfer from QDs to ammonia also impossible due to high position of lowest unoccupied molecular orbital (LUMO)^49^ in ammonia. It is probable that QDs PL quenching may be due to a long-range electronic-to-vibrational energy transfer^50^ from the QD to the NH-vibration in the ammonia molecule. Another possible reason for PL quenching is the formation of new local trap sites on the QD surface in the case of coordination of the two ammonia molecules on neighboring surface atoms similarly to the mechanism reported in the literature^51^.

After vapor treatment PL signal was almost instantaneously restored to its former values. It was confirmed by transient investigation of the PL signal. The PL decay parameters of samples during treatment are presented in Supporting Information (Table S11). For both samples under NH_3_ treatment average PL lifetime together with intensity decreased by ≈25% and then restored to the initial values. In addition, we carried out the experiment on the CdSe QD film formed by drop-casting the colloidal solution. Under vapor treatment of the sample its PL intensity has also decreased but only by the factor of 1.3. By contrast to the superstructures, the PL signal restored only in 20 minutes after the treatment. For detail see S12 in Supporting Information. Thus, it is clear that formed porous CdSe QD superstructures (C1 and C2) compared to CdSe QD film possess higher sensitivity with restored optical properties without a purifying procedure, e.g. degassing, which facilitates reusable applications of this material for fast detecting of analytes.

## Methods

### Experimental setup

SEM images of the obtained structures were taken using electron microscope Merlin (Zeiss). The luminescent images together with corresponding spectra were produced by laser scanning confocal microscope LSM-710 (Zeiss) equipped with 20× objective with NA = 0.4. The transient analysis of photoluminescence (PL) was carried out using the MicroTime 100 (PicoQuant) setup equipped with 100x objective with NA = 0.95. The excitation source in both cases was the diode laser operating at 405 nm.

### QD Synthesis

The preparation of alloyed 7.0 ± 1.0 nm QDs was achieved by the one-pot synthesis with slight modification according to Bae *et al*.^52^ 4.0 ± 0.5 nm CdSe QDs were synthesized according to previously reported procedure^53^. For details see Supporting Information (S1).

**Figure 5:**
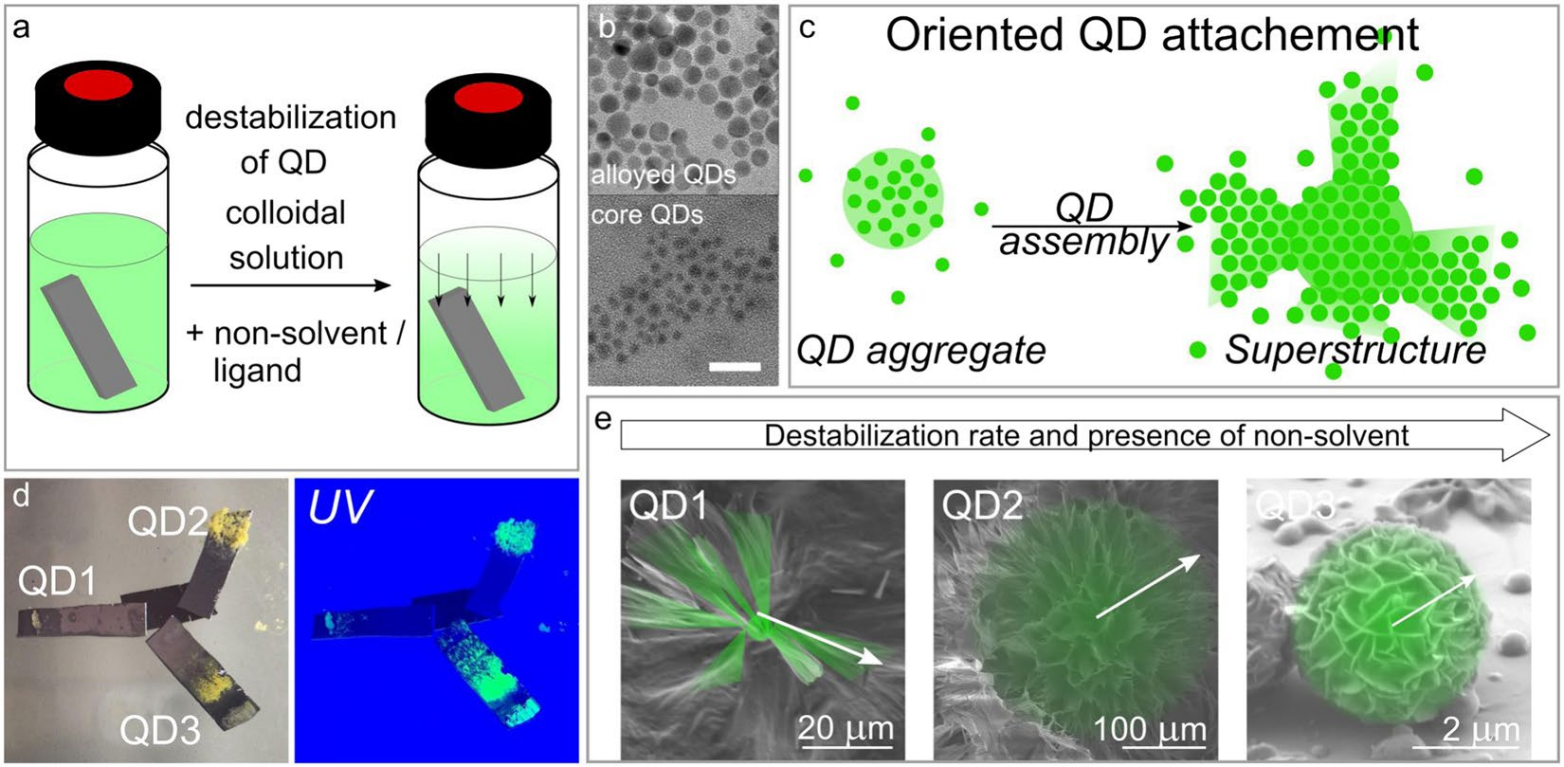
Preparation of the superstructures: (**a**) general scheme of a destabilization process; (**b**) TEM images of alloyed Cd_1−*x*_Zn_*x*_Se_1−*y*_S_*y*_/ZnS (upper panel) and core CdSe (lower panel) QDs, scale bar is 20 nm; (**c**) schematic presentation of superstructure formation by QDs; (**d**) photographs of QD1–QD3 samples under visible and UV light, (**e**) formation of different types of structures in dependence of destabilization rate and non-solvent presence. The arrows in (**e**) show the direction of superstructure growth.

To produce superstructures from QDs samples were prepared by different methods outlined below. Firstly, in the case of QD1–QD5, samples pre-cleaned Si substrates were placed in glass vials in inclined position. Then 500 *μ*l of alloyed Cd_1−*x*_Zn_*x*_Se_1−*y*_S_*y*_/ZnS QDs solution was added to each vial. The scheme of preparation methods and growth of superstructures are presented in Fig. 5. To fabricate the C1 and C2 samples, the pre-cleaned Si substrates were placed on the bottom of the glass vials, followed by the addition of 500 *μ*l of CdSe QD solution. To prepare the CdSe film a 20 *μ*l of stock QD solution was drop-casted on the Si substrate.

The QD1 sample was prepared by placement of an opened glass vial with the Si substrate and QDs solution in an empty closed weighing bottle. In this case, to create superstructure the method of the slow evaporation of QDs colloidal solution and following settling of QDs was applied^44^. To obtain QD2 and C1 the destabilization of colloidal solution by the non-solvent vapor was applied^45^. In this procedure, each opened glass vial with the Si substrate and alloyed QD or CdSe QD solution (for QD2 and C1, respectively) was placed in closed weighing bottle with 750 *μ*l of ethanol. The QD3 and C2 were formed by the solution destabilization method adopted from Talapin’ work^46^. Briefly, 100 *μ*l of isopropanol and 400 *μ*l of ethanol were added to the vial with 500 *μ*l of alloyed QD or CdSe QD solution (for QD3 and C2, respectively) and Si substrate. All components were carefully placed in layers to prevent mixing. Photographs of QD1–QD3 samples are presented in Fig. 5c. To prepare the QD4 we employed technique of self-assembly in the stabilizer excess^47^. In this experiment, the oleic acid in CHCl_3_ in ratio1:10 (V = 200 *μ*l) was added to the vial with QD solution and Si substrate. The reference sample (QD5) was formed by the deposition of Si substrate into a closed vial with 500 *μ*l of the initial QD solution. All vials were kept in a dark dry place at a temperature under 20 °C. It is worth to mention, that the superstructures formation on a substrate took different time periods. The QD3 was assembled after two week of slow destabilization. QD1 and QD2 samples took three weeks to self-assemble. The QD5 was obtained after one month. The QD4 was stable even after a month. When the temperature was lowered to 3–5 °C, the structures of QDs and oleic acid precipitated in the solution, and did not dissolve after increasing the temperature to 20 °C. C1 and C2 samples also took three weeks to self-assemble.

### Experiment with ammonia vapor

The cotton swab moistened with a 5% aqueous ammonia solution was placed in proximity to QD superstructures that roughly correspond to exposing of QDs with 100 ppm ammonia vapors. PL signal was registered during QD structures treatment by ammonia vapor using a laser scanning confocal microscope.

## Conclusions

We developed the preparation of highly porous luminescent material based on colloidal QDs. The morphology of structural elements depends on the destabilization rate of QD solution. It was found that the large flat spiky structures can be formed by the slow destabilization while solution evaporates, as it was observed for QD1 sample. The presence of the non-solvent vapor in this case promotes the formation of bulk flower-like structures with sizes of hundreds of microns (QD2 sample). By varying the destabilization rate in the presence of the non-solvent smaller structures can be formed with different shape: from spiky flowers to smooth spheres, which were found in the QD3 sample. The change in the surfactant molecule to QD molar ratio did not lead to the formation of desirable bulk porous structures.

Optical properties of superstructures formed remains almost the same compared to the parent QD solution. Decrease in PL lifetime for shorter components was observed for all samples, with an increase of longer lifetime amplitude in the case of QD2 and QD5 samples. This can be attributed to the appearance of QDs interactions within bulk ensembles formed. The comparison of optical properties with morphology of superstructures revealed that the preparation method with non-solvent destabilization is suitable for producing a material for sensor applications. It was confirmed on the porous superstructures formed by the core type CdSe QDs sensitive to the ammonia vapor. The 4-fold decrease in PL signal was observed for the best sample. It is worth to mention, that the optical properties were restored to the initial values, thus enabling to reuse such material without additional purification procedures. Importantly, these preparation approaches can be applied to nanoparticles of different nature along with their mixtures. Thus, these methods are expected to induce the rapid development of nanostructured porous materials with improved and controlled luminescent properties and morphology for new advanced optical sensor devices.

## Electronic supplementary material

Supplementary Information
