# Supplementary material for: Porous flower-like superstructures based on self-assembled colloidal quantum dots for sensing
Source: Sci Rep. 2019 Jan 24;9:617. doi: 10.1038/s41598-018-36250-1 (PMC6346065; doi:10.1038/s41598-018-36250-1)
Supplement: Supplementary file 1 — Supplementary Information [file 41598_2018_36250_MOESM1_ESM.pdf]

Supporting Information

Porous flower-like superstructures based on self-assembled colloidal quantum dots for sensing

Stepanidenko E.A.[1], GromovaYu.A.[1], Kormilina T.K.[1], Cherevkov S.A.[1], Kurshanov D.A.[1], Dubavik A.[1], Baranov M. A.[1], Medvedev O. S.[2], Fedorov A.V.[1], Gun’ko Y.K.[1,3], Ushakova E.V.[1] and Baranov A.V.[1]

[1] ITMO University, Saint Petersburg, 197101, Russia

[2] Saint-Petersburg State University, Saint Petersburg, 199034, Russia

[3] School of Chemistry and CRANN, Trinity College Dublin, Dublin 2

S1. Synthesis of $Cd_{1-x}Zn_xSe_{1-y}S_y$/ZnS and CdSe quantum dots and their optical properties

1. Chemicals

Cadmium oxide (CdO, 99.99 %), zinc oxide (ZnO, 99.9 %, powder), sulfur (99.9 %, powder), selenium (99.99%, powder), sulphur (99.99%, powder), trioctylphosphine (TOP, 90 %), oleic acid (OlAc, 90 %), 1-octadecene (1-ODE, 90 %), oleylamine (OLAm,70%)were used as purchased from Aldrich.

2. Synthesis

$Cd_{1-x}Zn_xSe_{1-y}S_y$/ZnS QDs. The preparation of QDs was passed by one-pot synthesis with slight modification according to Bae et al.[Bae, W. K.; Char, K.; Hur, H.; Lee, S. Single-Step Synthesis of Quantum Dots with Chemical Composition Gradients. Chem. Mater. 2008, 20 (2), 531–539]. Briefly, 0.2 mmol of CdO and 4 mmol of ZnO were placed with 5 ml of OlAc and 15 ml of 1-ODE in 100 ml of flask, heated to 150 °C and evacuated for 30 min. The reaction vessel was keeping under Ar atmosphere condition and heated up to 300°C resulting clear mixed solution of $Cd(oleate)_2$ and $Zn(oleate)_2$ was acquired. At the temperature 300°C solution of 0.1 mmol of Se and 4 mmol of S dissolved in 2 ml of TOP was quickly injected into the reaction flask. To produce QDs with chemical composition gradient the reaction was continued at that temperature for 10 min. Then the temperature of obtained solution was reduced to room temperature to stop the reaction. To purified obtained QDs them were dispersing in chloroform and then precipitating with excess amount of acetone. This procedure was repeated several times. Finally, purified QDs were dispersed in chloroform with addition of small amount of OlAc. These initial alloyed QDs solution was used for further self-assembly experiments. Optical spectra of $Cd_{1-x}Zn_xSe_{1-y}S_y$/ZnS quantum dots and its TEM images represent on Fig. S1.

CdSe cores. The QDs were synthesized according to ref. Protière, M.; Nerambourg, N.; Renard, O.; Reiss, P. Rationaldesign of the gram-scale synthesis

of nearly monodisperse semiconductor nanocrystals. Nanoscale Res. Lett.2011, 6, 472.

Briefly, the syntheses of all precursors and final quantum dots were conducted under argon using standard Schlenk-line techniques:

i) Se-precursor: 0.19 g (0.4 M) of selenium (Se) was added to a 25 mL two-necked round-bottomed flask. The flask was sealed, evacuated, and then kept under argon. 5 mL of TOP was added to the selenium powder. The solution was sonicated at room temperature until a clear solution.

ii) Cd-precursor: 0.051 g (0.4 mmol) of CdO, 2.845 g (10 mmol) of OlAc, 13 mL (~42.5 mmol) of OLAm, and 9.3 mL of ODE were added to a 100 mL three-necked flask. The flask was then evacuated for 1 hour at 90 C, after that it was slowly heated to 250 °C under argon flow.

iii) CdSe synthesis: when the temperature of the solution ii (Cd-precursor) reached 250 was completely dissolved (clear solution), the TOP−Se solution was injected swiftly into the reaction flask. After the injection, the nanocrystals were heated further to grow for different time intervals (up to 30 min) depending on the desired nanocrystal size. After the synthesis the flask was allowed to cool to the room temperature.

The QDs were centrifuged after adding acetone. The precipitate containing QDs was redispersed by small volume of non-polar solventwith addition of small amount of OlAc. The concentration of QD solution was 1.74×10^{-7} M.

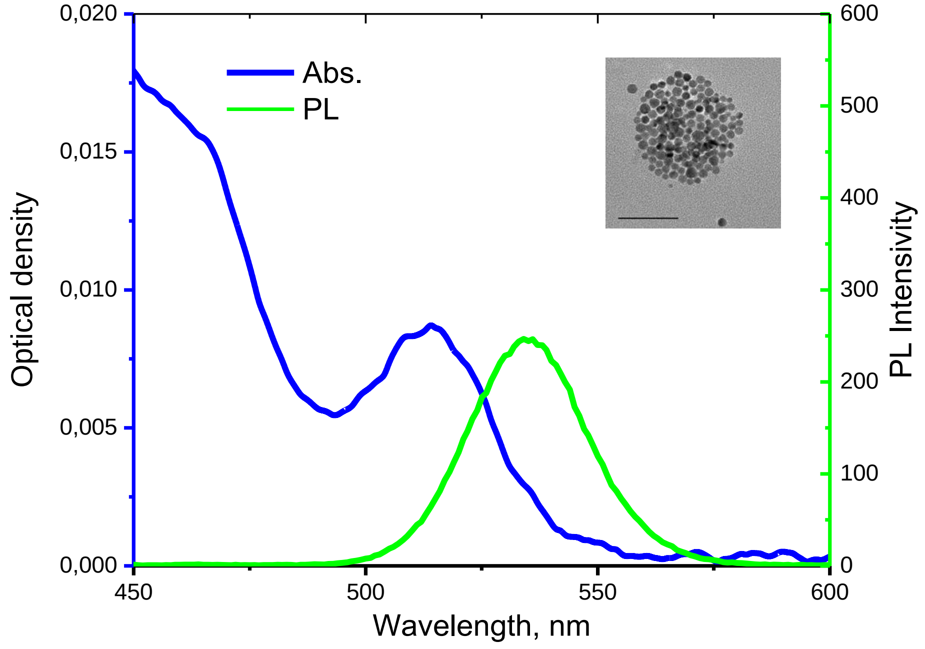

Figure S1.1. Optical spectra of initial solution of $Cd_{1-x}Zn_xSe_{1-y}S_y/ZnS$ quantum dots. Sketch – TEM images of $Cd_{1-x}Zn_xSe_{1-y}S_y/ZnS$ quantum dots. Scale bar is of 50 nm.

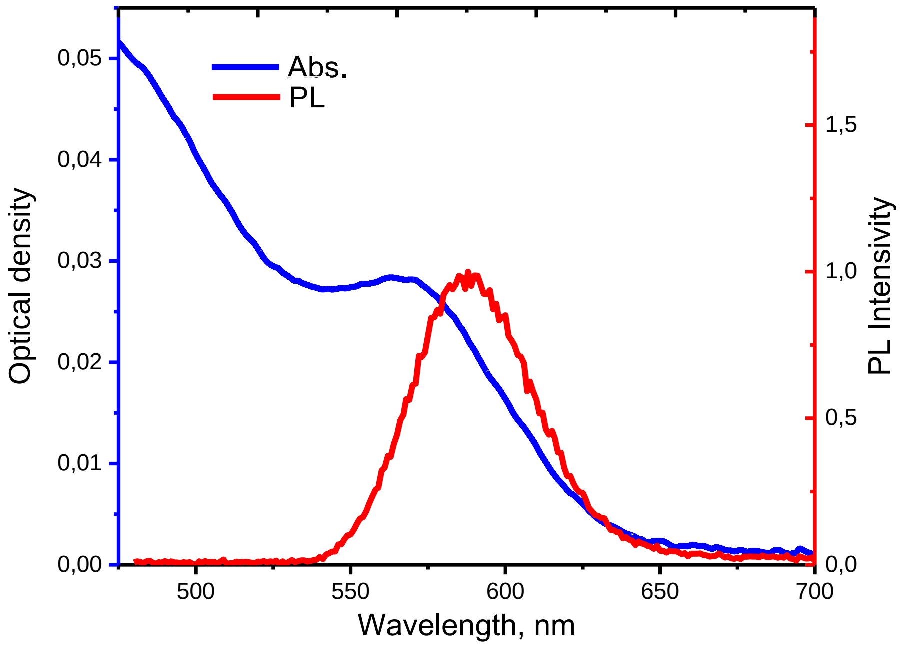

Figure S1.2. Optical spectra of initial solution of CdSe QDs

S2. SEM images of sample QD1 formed by alloyed QDs

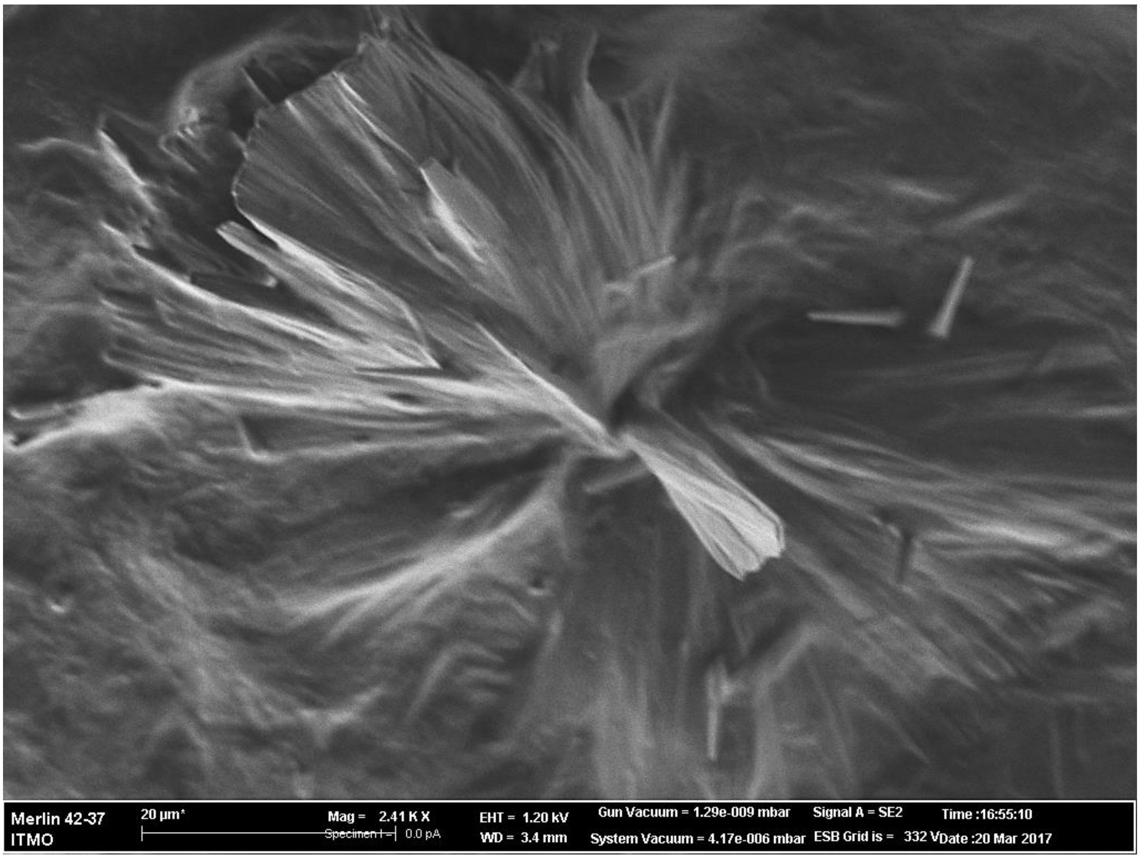

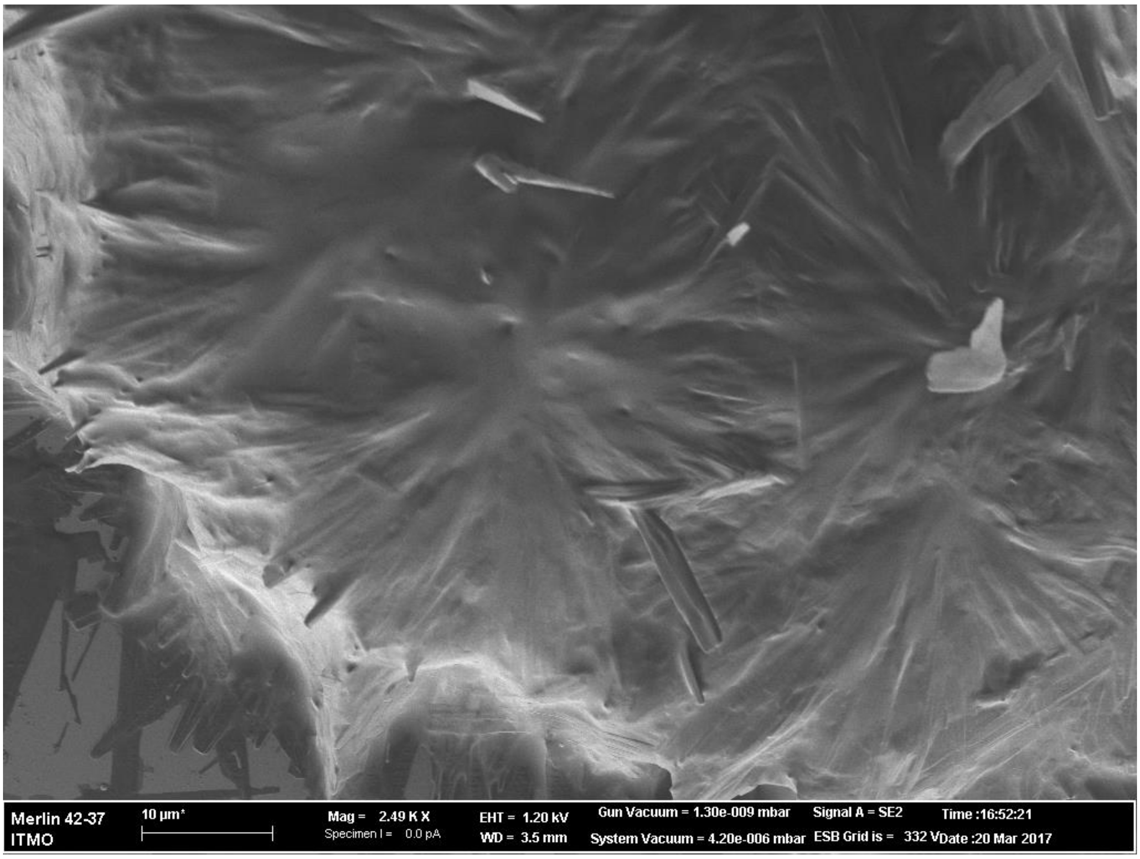

Figure S2.1. SEM images of the superstructures formed by $Cd_{1-x}Zn_xSe_{1-y}S_y/ZnS$ QDs in sample QD1

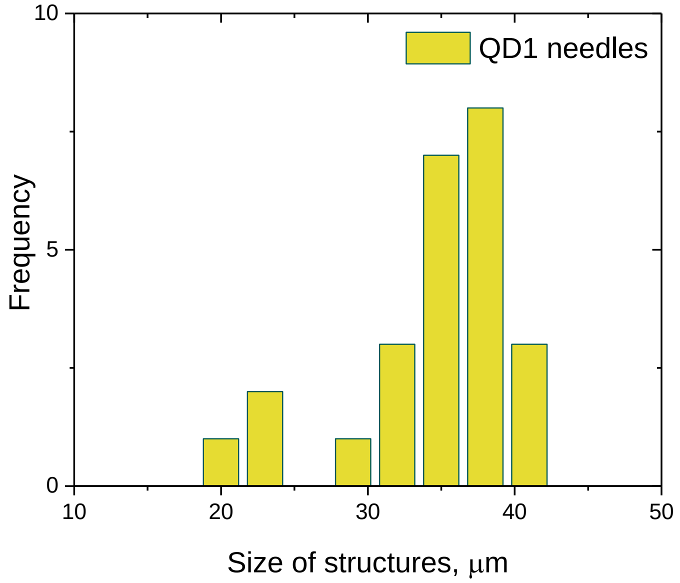

Figure S2.2. Histograms of size distribution for size of typical structures formed in QD1

S3. SEM images of sample QD2 formed by alloyed QDs

Bulk porous structure made from spiky flower (bottom part of substrate)

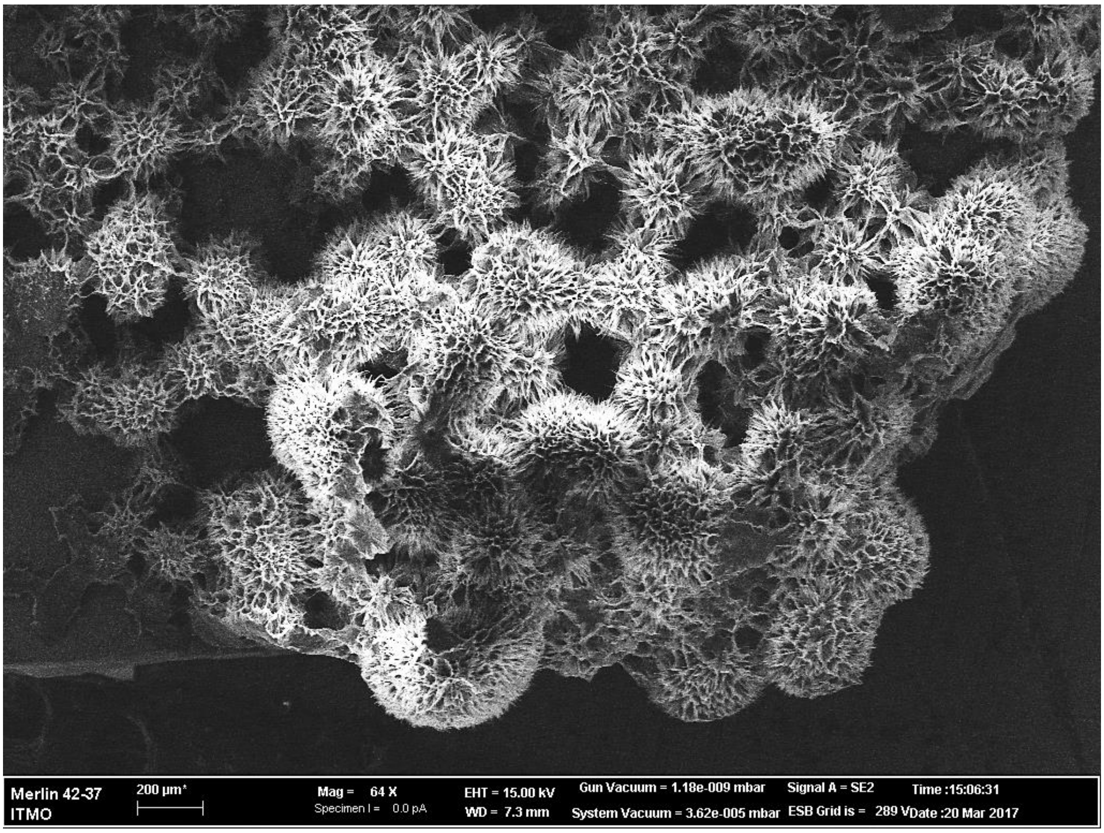

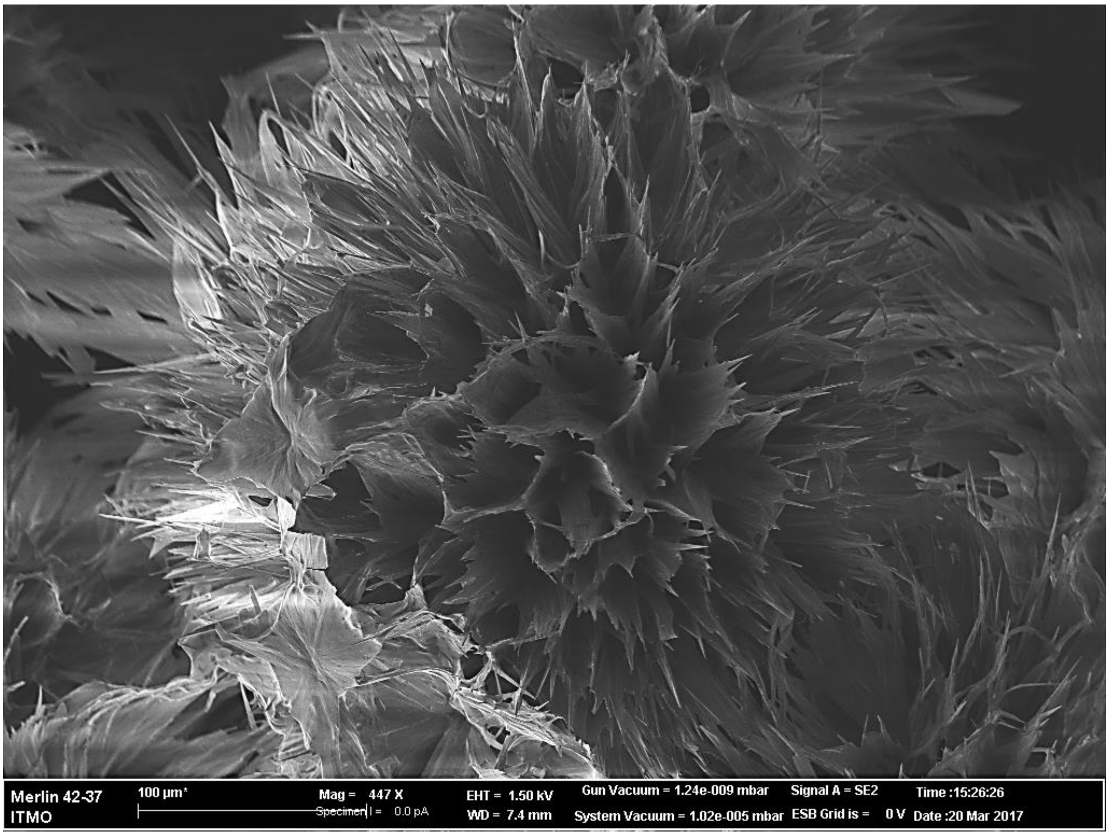

Figure S3.1. SEM images of the superstructures formed by $Cd_{1-x}Zn_xSe_{1-y}S_y/ZnS$ QDs in sample QD2

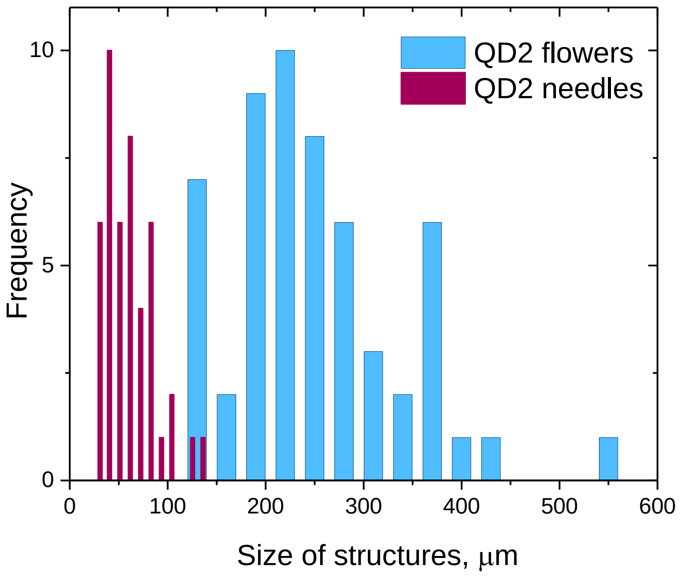

Figure S3.2. Histograms of size distribution for size of typical structures formed in QD2

S4. SEM images of sample QD3 formed by alloyed QDs

Spiky flowers and spheres

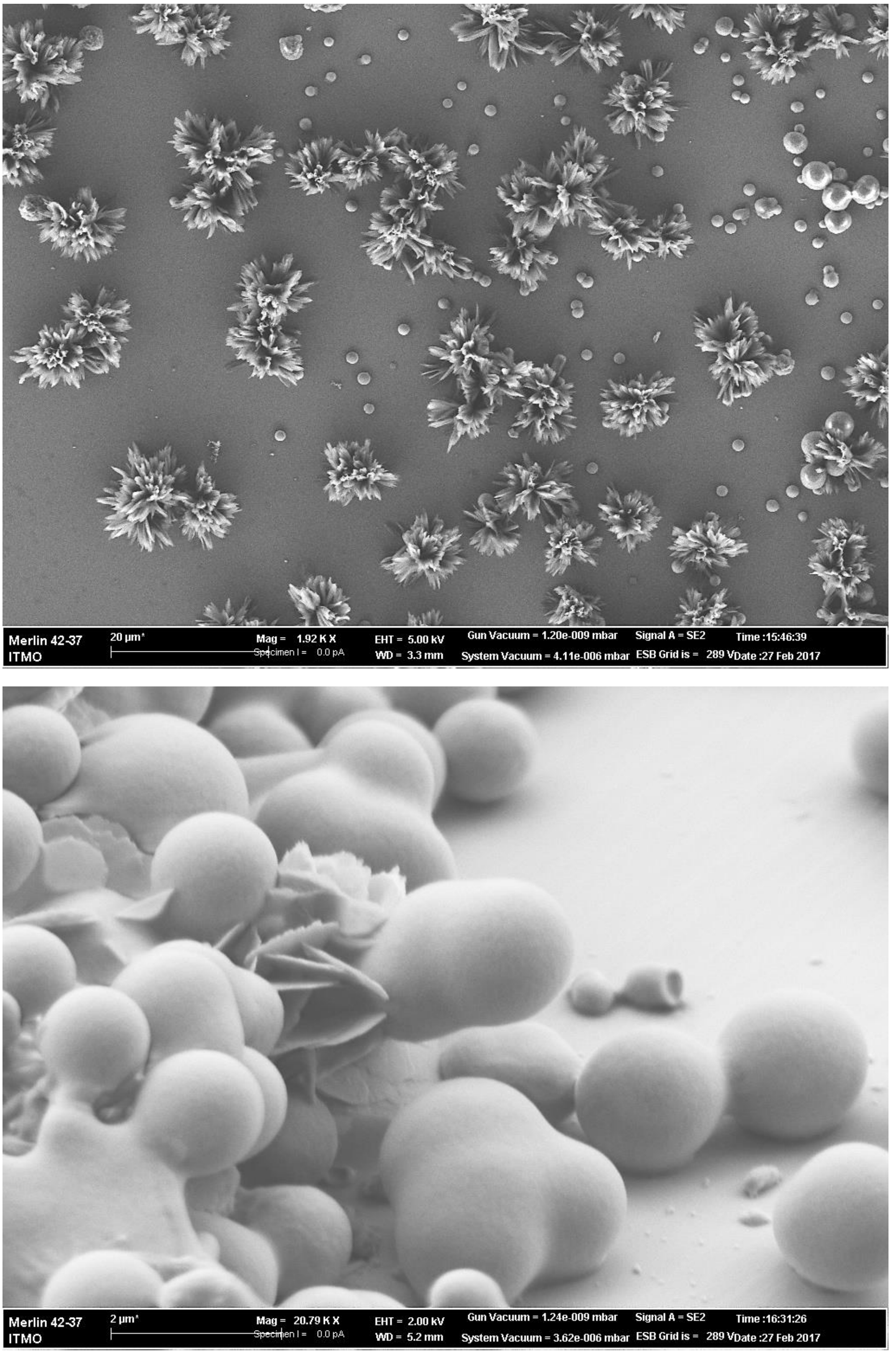

Figure S4.1. SEM images of the superstructures formed by $Cd_{1-x}Zn_xSe_{1-y}S_y/ZnS$ QDs in sample QD3

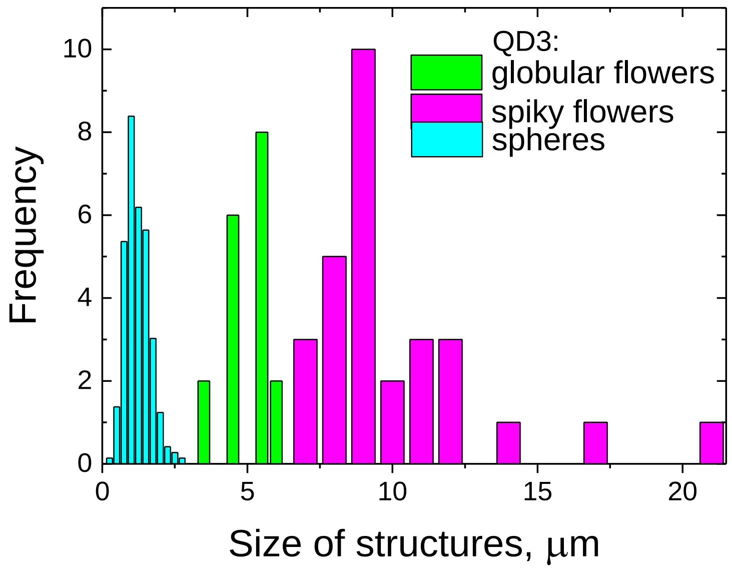

Figure S4.2. Histograms of size distribution for size of typical structures formed in QD3

S5. Optical images of sample QD4 formed by alloyed QDs

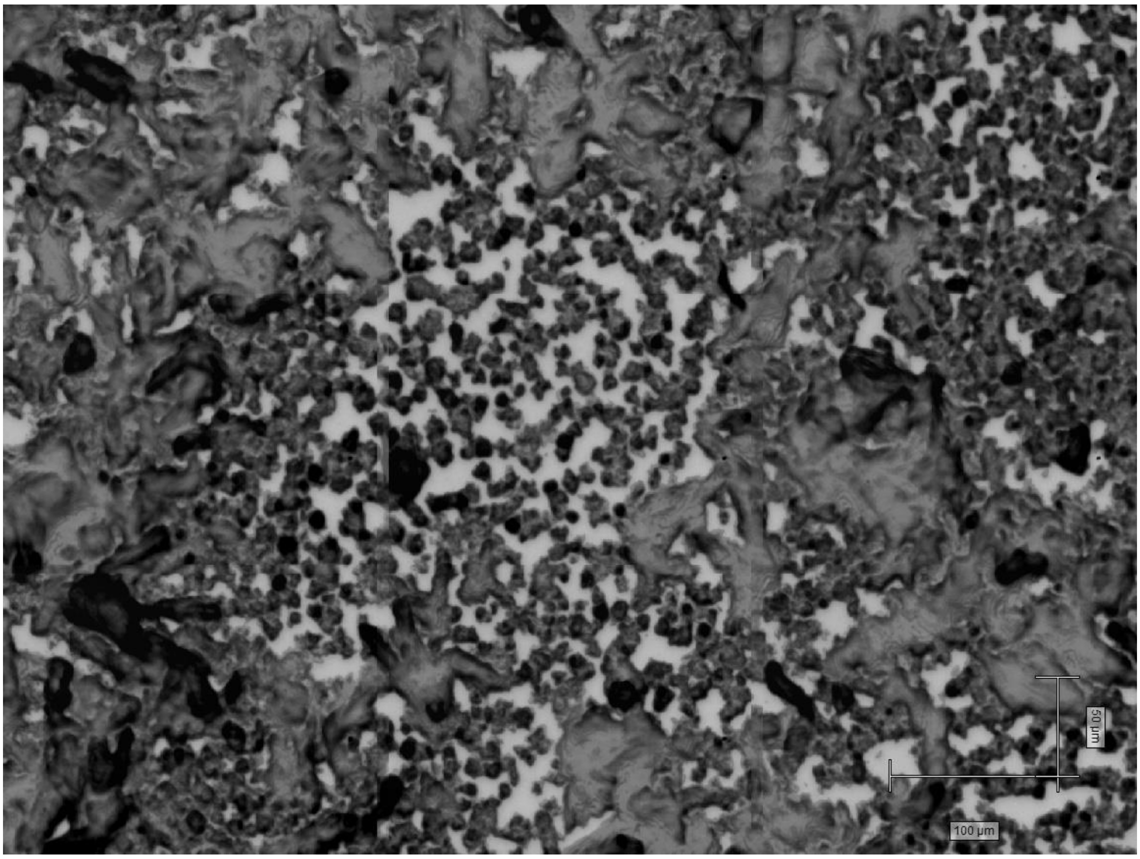

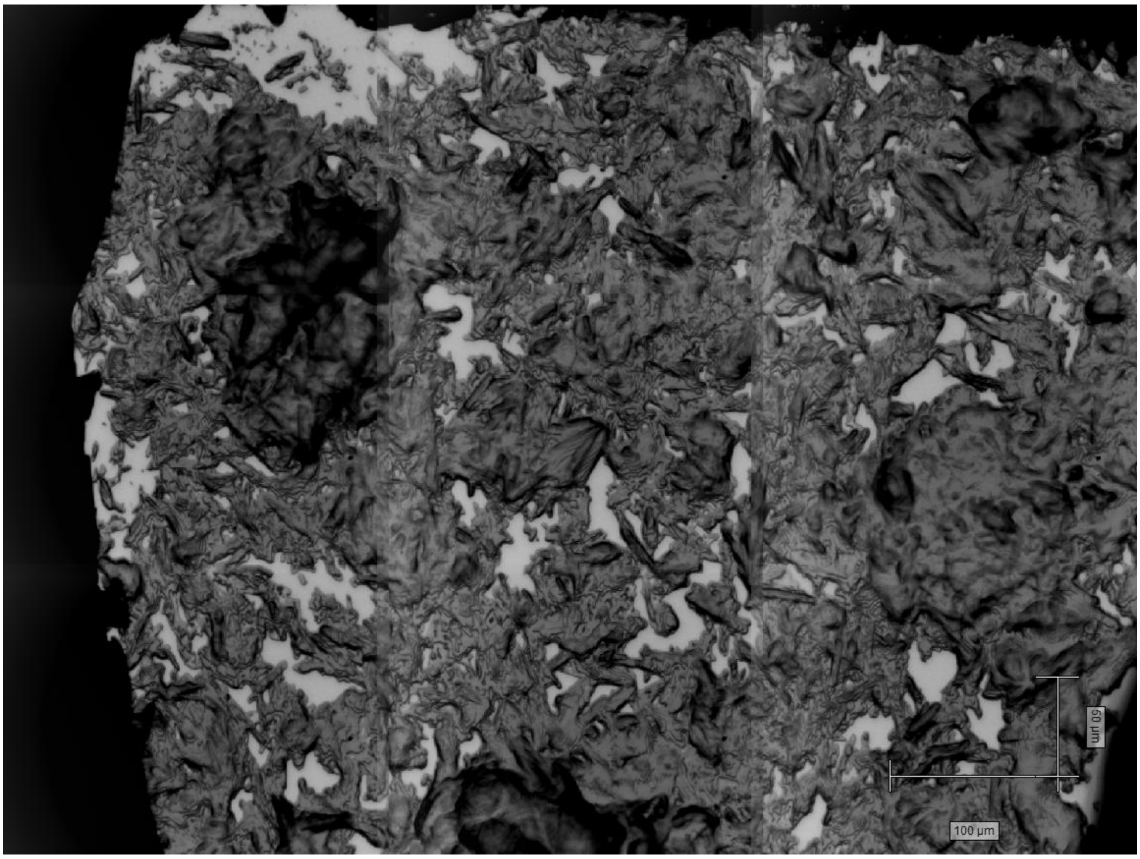

Figure S5.1. Optical images of the sample QD4 formed by $Cd_{1-x}Zn_xSe_{1-y}S_y/ZnS$ QDs

S6. SEM images of sample QD5 formed by alloyed QDs

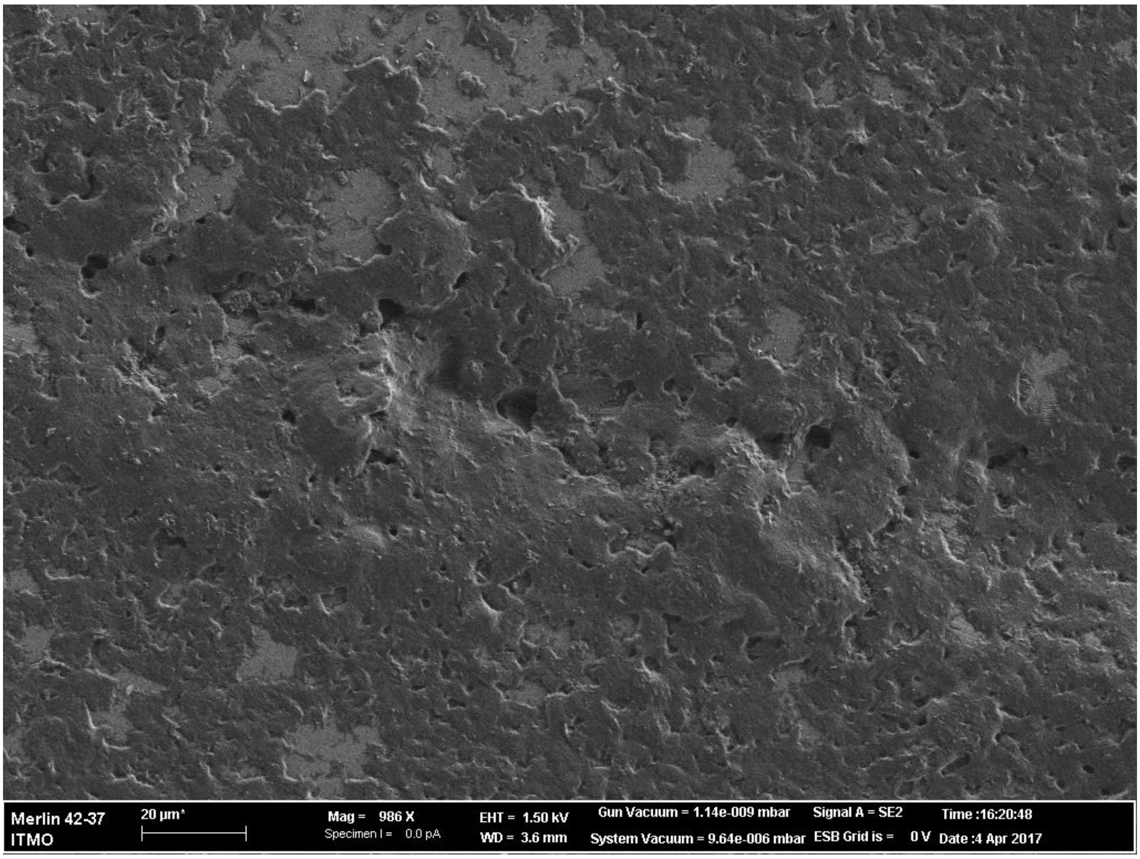

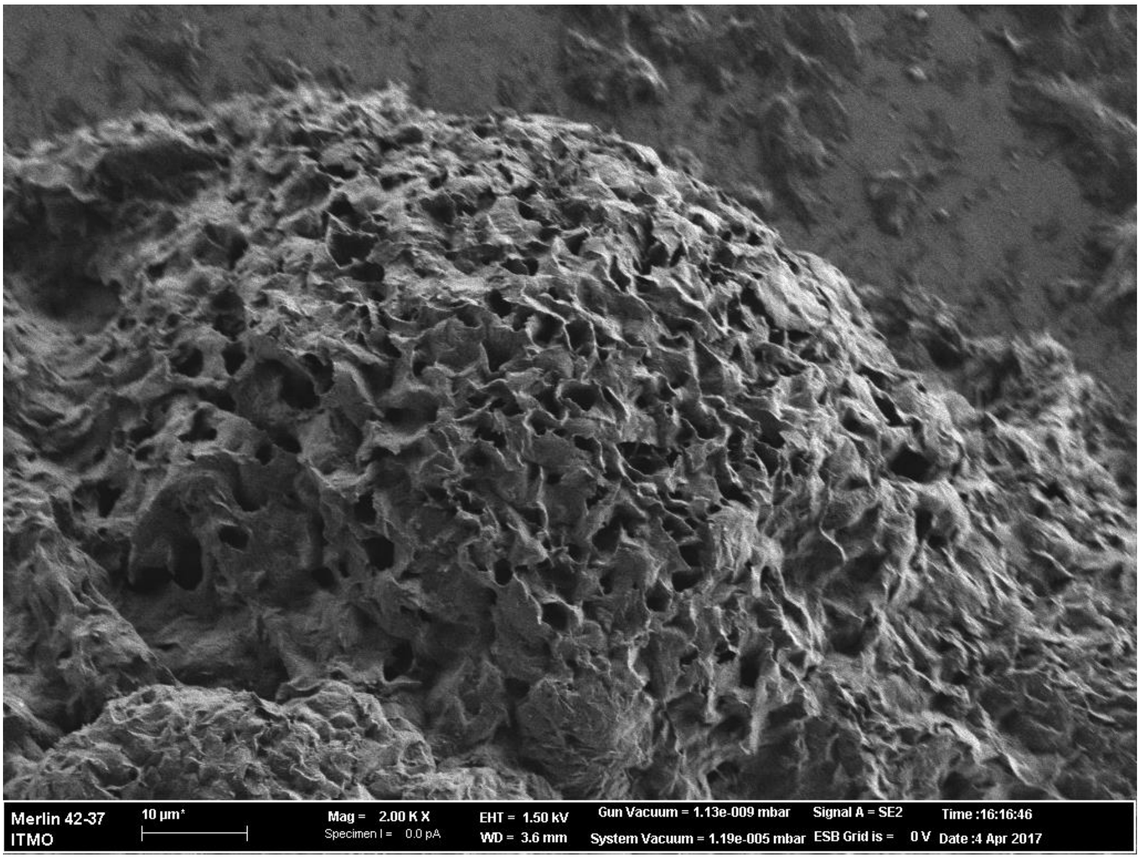

Figure S6.1. SEM images of the sample QD5 formed by $Cd_{1-x}Zn_xSe_{1-y}S_y/ZnS$ QDs

S7. Elemental analysis of typical superstructure formed by alloyed QDs

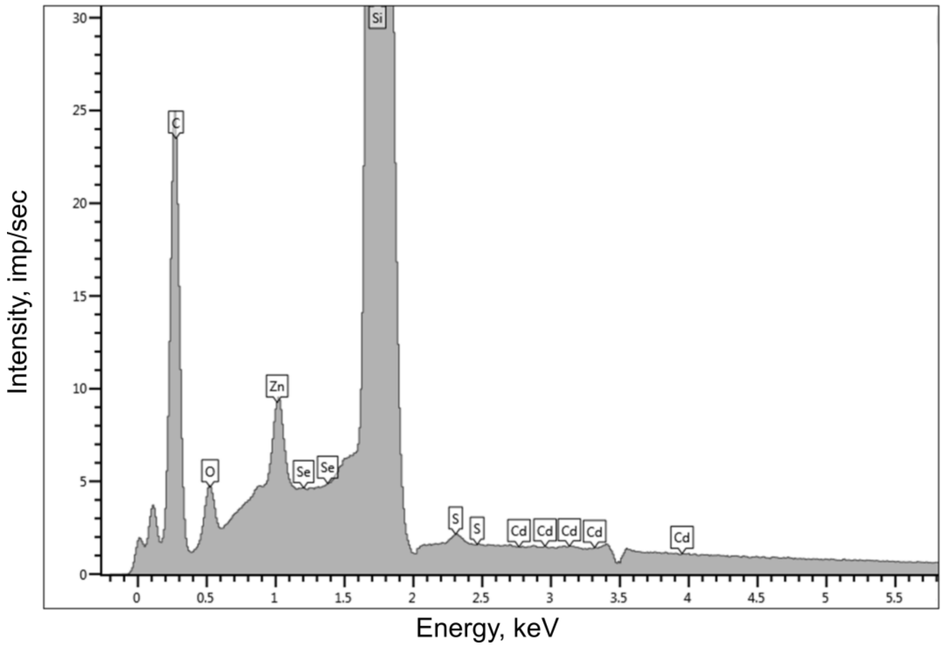

Figure S7. Energy-dispersive X-ray spectrum of typical superstructure formed by $Cd_{1-x}Zn_xSe_{1-y}S_y/ZnS$ QDs

S8. Optical properties of QD1-QD5 samples based on alloyed QDs

(a) (b)

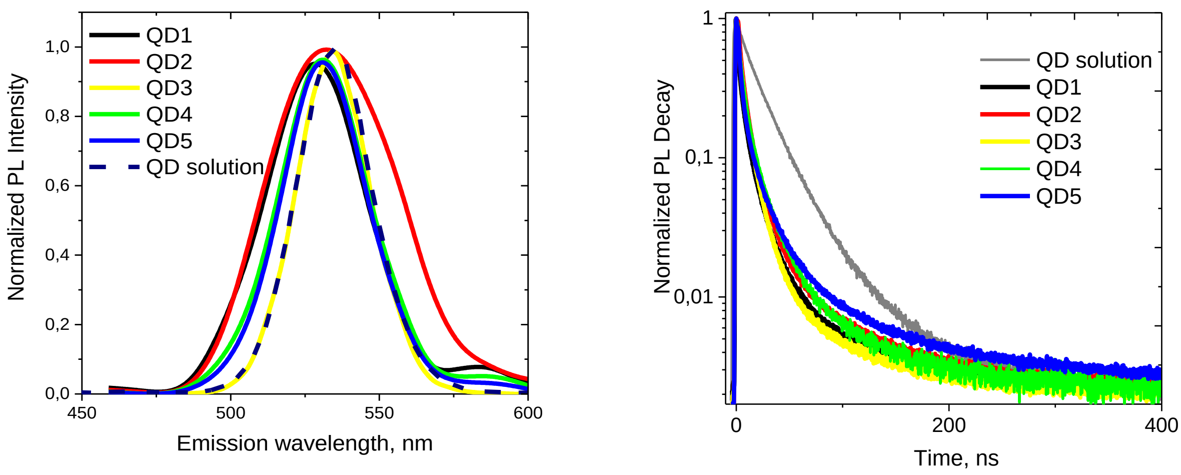

Figure S8.1 PL spectra (a) and PL decay (b) QD1 – QD2 for samples and initial $Cd_{1-x}Zn_xSe_{1-y}S_y/ZnS$ QD solution

Table S8. PL decay parameters

Sample	A_1	τ_1, ns	A_2	τ_2, ns	A_3	τ_3, ns	τ_{av}, ns
QD solution	18915	15.4	13803	31.9	217	153.9	30.9
QD1	36965	3.9	25575	13.6	1938	65.0	21.7
QD2	57793	5.8	15096	20.8	941	144.3	35.7
QD3	46129	4.6	30479	11.9	1520	66.5	17.8
QD4	26097	4.1	10186	16.1	274	150.0	29.6
QD5	35754	4.5	16309	22.2	1057	169.4	55.7

S9. Sensor on alloyed QDs

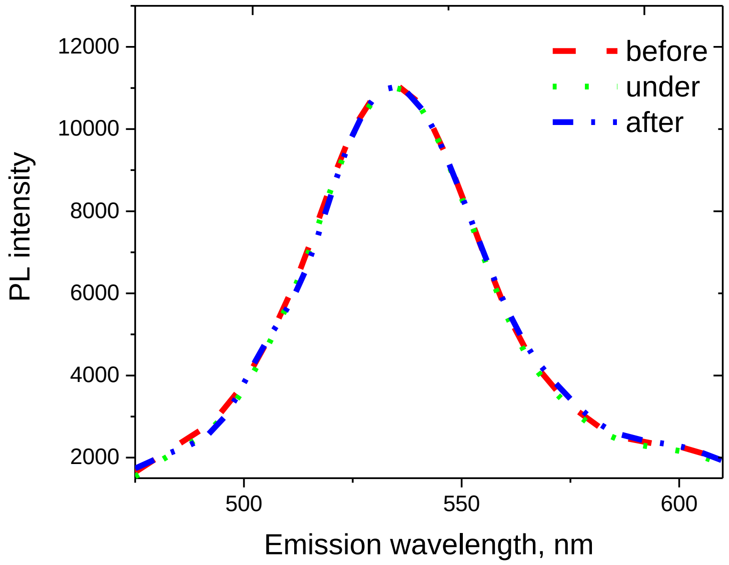

Figure S9.1. Sensing properties of sample QD2 formed by $Cd_{1-x}Zn_xSe_{1-y}S_y/ZnS$ QDs

S10. SEM images of samples C1 and C2 formed by core QDs

a) Porous structure of sample C1

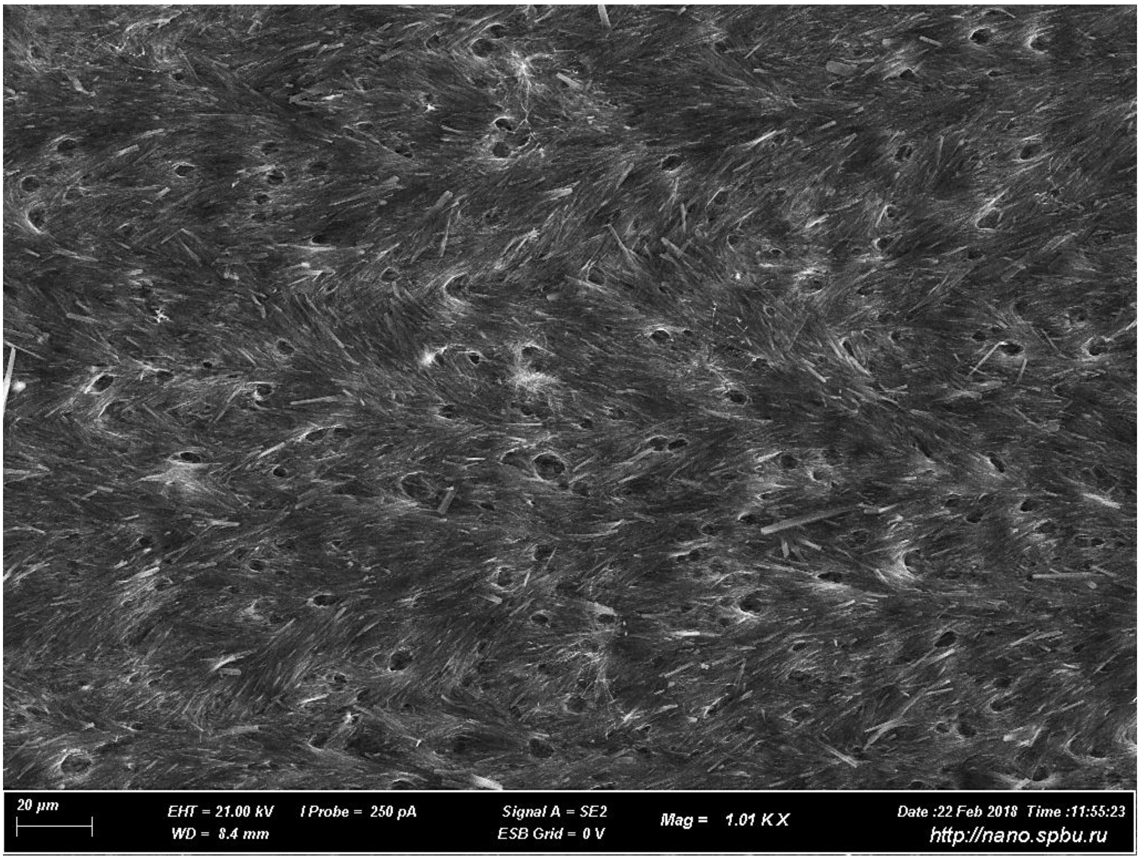

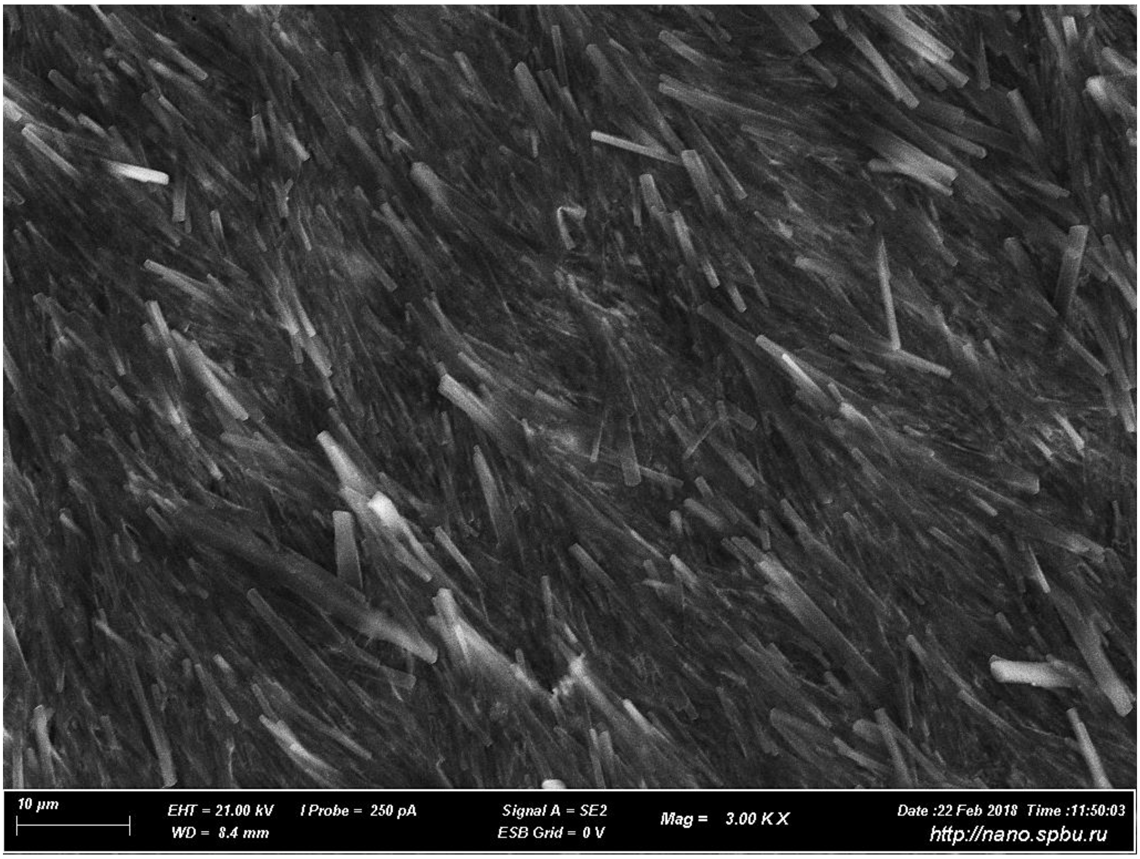

b) Sample C2. Spiky Microflowers and Globular microflowers

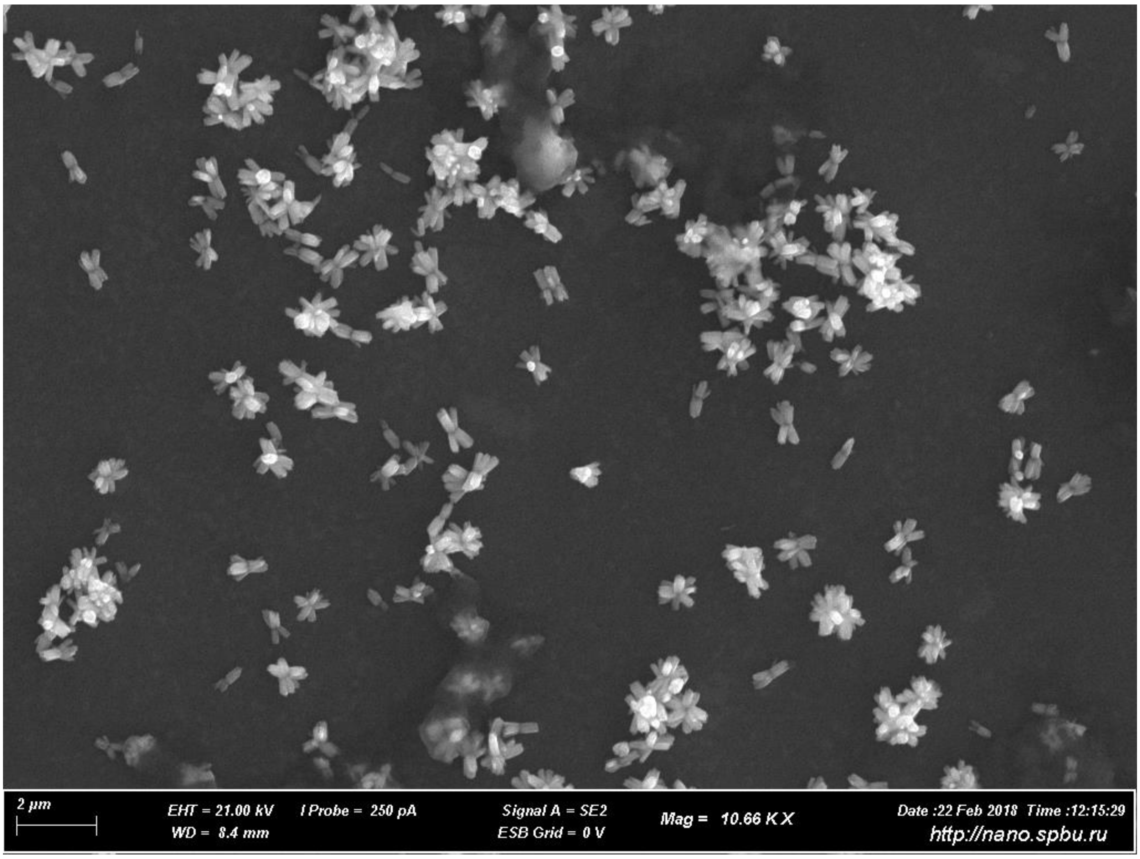

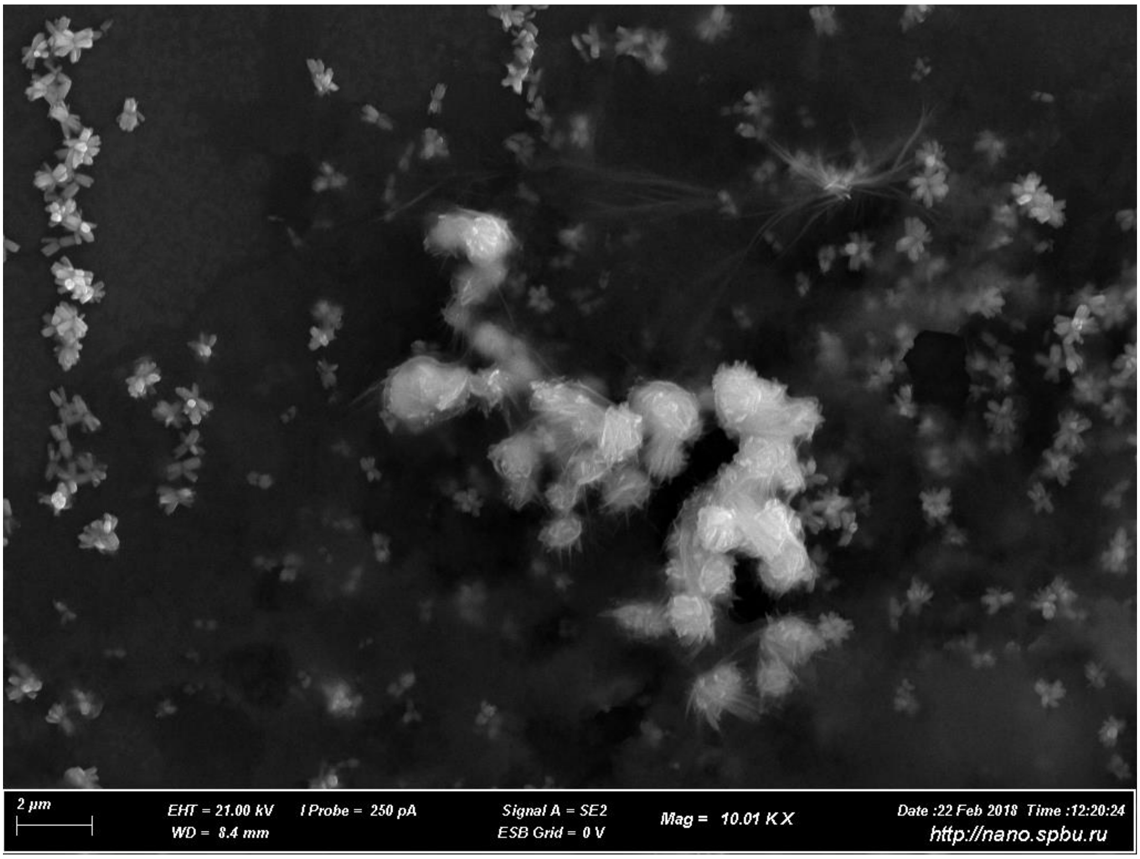

Figure S10.1. SEM images of the superstructures formed by CdSe QDs in samples a) C1 and b) C2

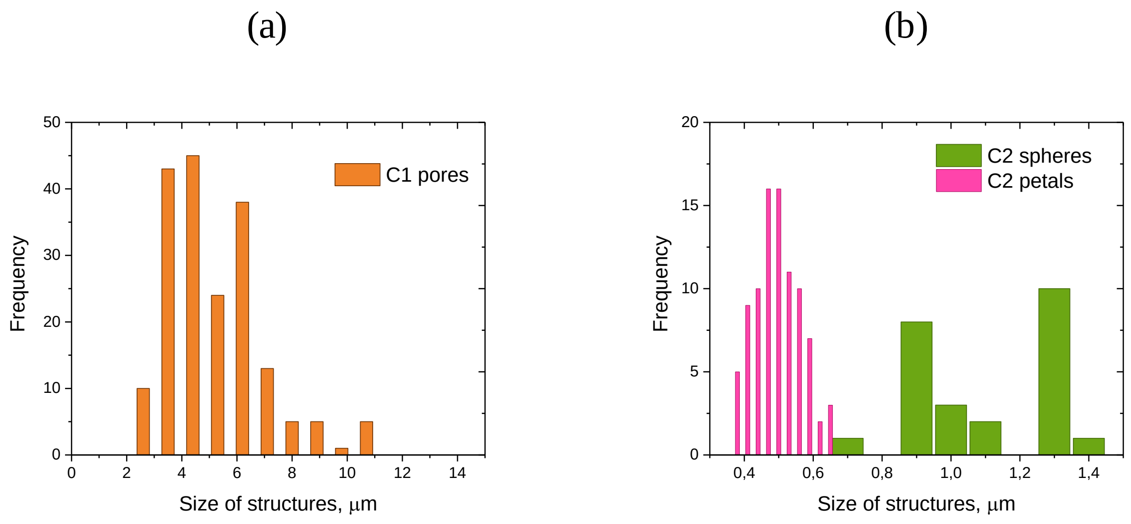

Figure S10.2. Histograms of size distribution for size of typical structures formed in a) C1 and b) C2

S11. Sensing properties of superstructures based on CdSe QDs

(a) (b)

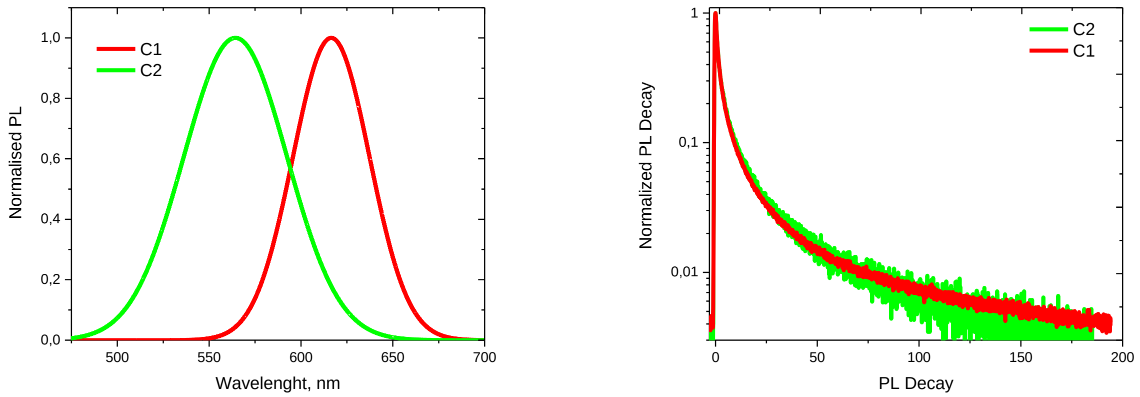

Figure S11.1 PL spectra (a) and PL decay (b) for C1 and C2 samples formed by CdSe QDs

(a) (b)

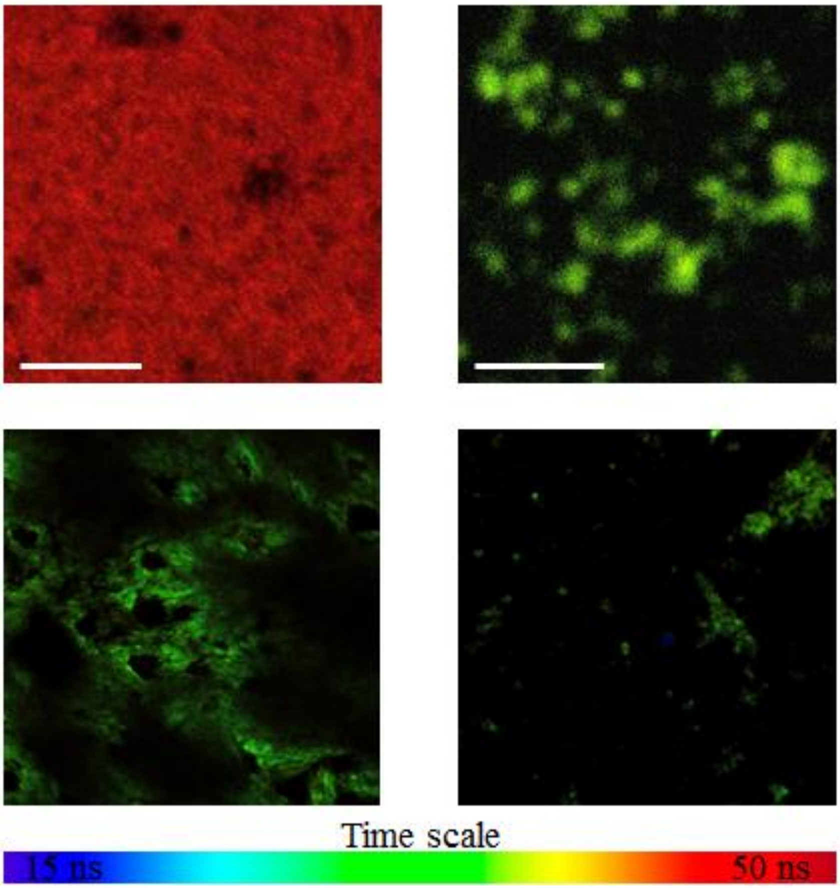

Figure S11.2. PL images (upper panel) and FLIM (lower panel) for a) C1 and b) C2. Scale bare: 50 µm, FLIM size: 80.00 µm x 80.00 µm

(a) (b)

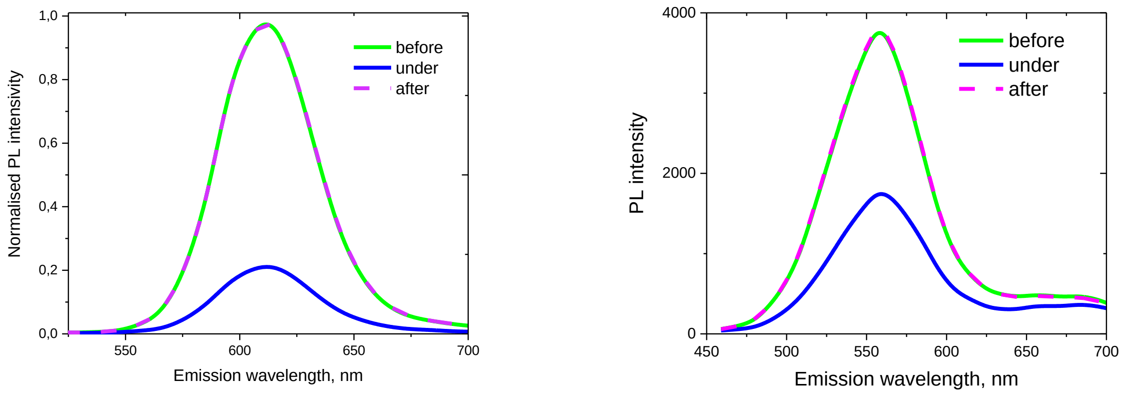

Figure S11.3. Sensing properties of samples a) C1 and b) C2

Table S11. Average PL lifetimes of treated samples formed by CdSe QDs

Sample name	Average PL lifetime, ns		
	Before NH_3	Under NH_3	AfterNH_3
C1	18.2±0.5	14.0±0.7	17.8±0.5
C2	19.0±0.5	15.8±0.7	18.2±0.5

S12. Sensing properties of the film based on CdSe QDs

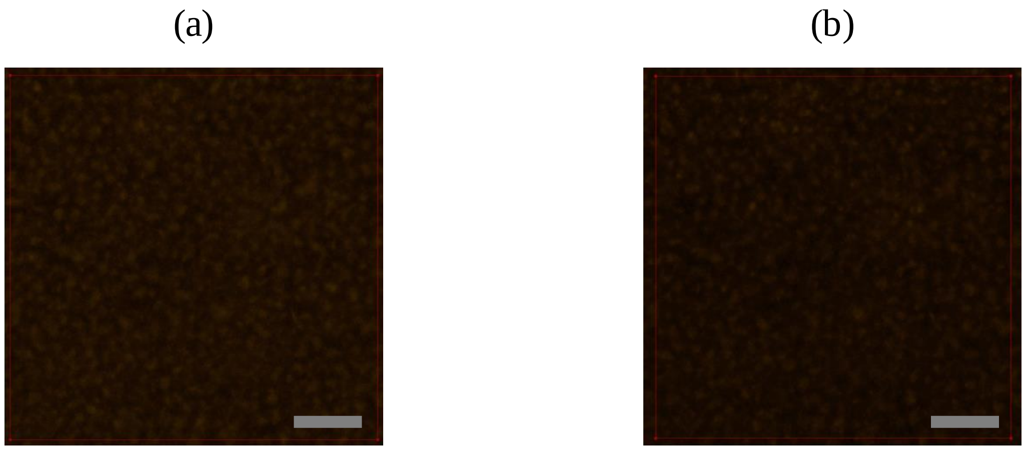

Figure S12.1. PL images of the film based on CdSe QDs: a) initial PL, b) PL under NH_3 treatment. Scale bare: 25 μm

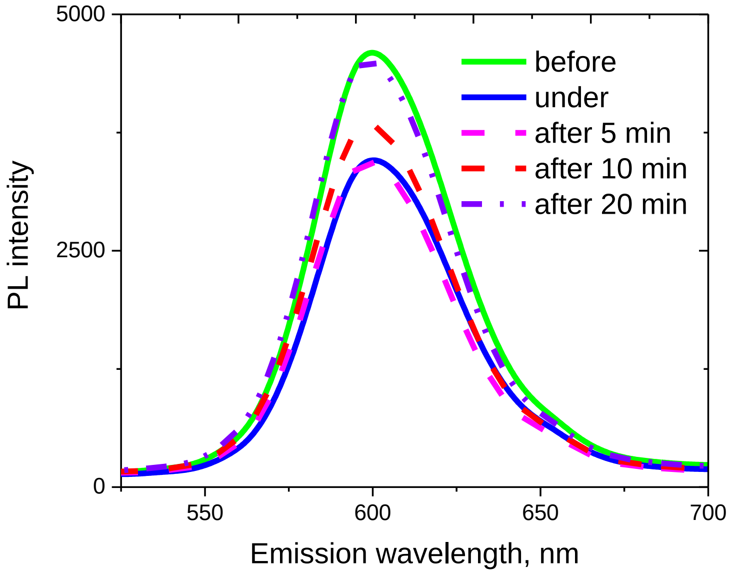

Figure S12.1. Sensing properties of the film based on CdSe QDs

Table S12. PL response of the film based on CdSe QDs under NH_3 treatment

	under NH_3 treatment	after 5 min	after 10 min	after 20 min
% of the initial PL intensity of the film	74	76	84	99
